# A rationally designed fluorescence probe achieves highly specific and long‐term detection of senescence in vitro and in vivo

**DOI:** 10.1111/acel.13896

**Published:** 2023-06-13

**Authors:** Li Hu, Chanjuan Dong, Zhe Wang, Shengyuan He, Yiwen Yang, Meiting Zi, Huiqin Li, Yanghuan Zhang, Chuanjie Chen, Runzi Zheng, Shuting Jia, Jing Liu, Xuan Zhang, Yonghan He

**Affiliations:** ^1^ Key Laboratory of Healthy Aging Research of Yunnan Province, Kunming Institute of Zoology Chinese Academy of Sciences Kunming China; ^2^ State Key Laboratory of Genetic Resources and Evolution, Kunming Institute of Zoology Chinese Academy of Sciences Kunming China; ^3^ University of Chinese Academy of Sciences Beijing China; ^4^ Drug Discovery & Development Center, Shanghai Institute of Materia Medica Chinese Academy of Sciences Shanghai China; ^5^ School of Chinese Materia Medica Nanjing University of Chinese Medicine Nanjing China; ^6^ Laboratory of Molecular Genetics of Aging and Tumor, Medical School Kunming University of Science and Technology Kunming China

**Keywords:** aging, fibrosis, near‐infrared probe, senescent cells, β‐galactosidase

## Abstract

Senescent cells (SnCs) are implicated in aging and various age‐related pathologies. Targeting SnCs can treat age‐related diseases and extend health span. However, precisely tracking and visualizing of SnCs is still challenging, especially in in vivo environments. Here, we developed a near‐infrared (NIR) fluorescent probe (XZ1208) that targets β‐galactosidase (β‐Gal), a well‐accepted biomarker for cellular senescence. XZ1208 can be cleaved rapidly by β‐Gal and produces a strong fluorescence signal in SnCs. We demonstrated the high specificity and sensitivity of XZ1208 in labeling SnCs in naturally aged, total body irradiated (TBI), and progeroid mouse models. XZ1208 achieved a long‐term duration of over 6 days in labeling senescence without causing significant toxicities and accurately detected the senolytic effects of ABT263 on eliminating SnCs. Furthermore, XZ1208 was applied to monitor SnCs accumulated in fibrotic diseases and skin wound healing models. Overall, we developed a tissue‐infiltrating NIR probe and demonstrated its excellent performance in labeling SnCs in aging and senescence‐associated disease models, indicating great potential for application in aging studies and diagnosis of senescence‐associated diseases.

## Graphical abstract

Senescence is a driver of aging and various age‐related diseases. However, tracking and visualizing of senescence in vivo is very challenging. We have rationally developed a near‐infrared (NIR) fluorescent probe (XZ1208) that can be cleaved rapidly by ß‐galactosidase (ß‐Gal) and produces a strong fluorescence signal. XZ1208 achieved high specificity, sensitivity and a long‐term duration in labeling senescence without causing significant toxicities in multiple aging and senescence‐associated disease models.

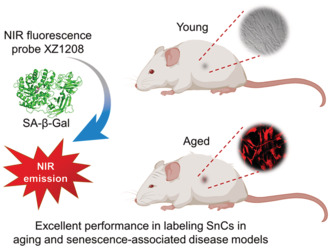

**Abbreviations**
ACN: acetonitrile
ALT: alanine transaminase
BLM: bleomycin
CTB: cathepsin B
CPDL: cumulative population doubling level
DAPI: 4',6‐diamidino‐2‐phenylindole
DCM: dicyanomethylene‐4H‐pyran
FA: folic acid
HDF: human dermal fibroblasts
HEL: human embryonic lung fibroblasts
HFD: high fat diet
HPLC: high‐performance liquid chromatography
ICT: intramolecular charge transfer
IR‐SnCs: irradiation‐induced senescent cells
MRI: magnetic resonance imaging
NIR: near‐infrared
PET: positron emission tomography
REP‐SnCs: replicatively senescent cells
ROIs: regions of interest
SA‐β‐Gal: senescence‐associated β‐galactosidase
SASP: senescence‐associated secretory phenotype
SnC: senescent cell
STZ: streptozotocin
TBI: total body irradiated

## 1 INTRODUCTION

Cellular senescence is the process by which cells enter a permanent state of irreversible cell‐cycle arrest (Hayflick, 1965). It can be caused by extensive replication or induced by diverse stimuli, such as oxidative stress, genotoxic damage, oncogene activation, epigenetic changes, and proteasome inhibition (Hernandez‐Segura et al., 2018). Cellular senescence can be a physiologically and pathologically relevant process depending on the situation (Herranz & Gil, 2018). Regarding the former, senescent cells (SnCs) play beneficial roles in tumor suppression and wound healing (Demaria et al., 2014; He & Sharpless, 2017). Regarding the latter, abnormal accumulation of SnCs is causally implicated in biological aging and various age‐related diseases (Childs et al., 2017). SnCs have been shown to exert their functions primarily through the secretion of senescence‐associated secretory phenotype (SASP) factors (Demaria et al., 2017).

Since the critical discovery that clearance of SnCs by genetic strategies can delay aging, ameliorate age‐related pathologies, and extend lifespan in mice (Baker et al., 2016), we and others have identified a series of targets for intervention in SnCs and developed several classes of pharmaceutical compounds that can selectively kill SnCs (termed senolytics) or suppress SASP (termed senomorphics) (He, Li, et al., 2020; Kirkland & Tchkonia, 2020; van Deursen, 2019). These compounds have been widely used to treat various senescence‐associated diseases and achieved great progress, with several senolytics now under clinical investigation (Chaib et al., 2022; Kirkland & Tchkonia, 2020). However, the development of SnC‐targeted interventions remains challenging, especially in terms of safety, specificity, and broad‐spectrum activity (Ge et al., 2021). One of the major obstacles is the lack of sensitive methods to selectively track SnCs (Yao et al., 2021). Tracking SnCs typically relies on the detection of markers and phenotypic characteristics of SnCs (Hernandez‐Segura et al., 2018), including expression of cell‐cycle inhibitors (p16 and p21), SASP factors, and senescence‐associated β‐galactosidase (SA‐β‐Gal) activity. Among them, SA‐β‐Gal is the most widely used marker for SnC detection (Lozano‐Torres et al., 2019). Human SA‐β‐Gal is a lysosomal exoglycosidase that removes galactose residues from substrates. SA‐β‐Gal can be detected in cells and fresh tissues using a colorimetric assay with X‐gal as a chromogenic substrate (Debacq‐Chainiaux et al., 2009). However, poor cell permeability of X‐gal makes it challenging to perform in vivo imaging. Although several new methods have been developed to detect SA‐β‐Gal (Sharma et al., 2021), such as bioluminescence, chemiluminescence, magnetic resonance imaging (MRI), single photoemission computed tomography, and positron emission tomography (PET), most have failed to achieve real‐time in situ non‐destructive detection (Sharma et al., 2021). In comparison, fluorescent probes show advantages over other detection methods due to their convenience, high sensitivity, and bioimaging ability (Li et al., 2020; Lozano‐Torres et al., 2017; Sharma et al., 2021; Zhang et al., 2016). Furthermore, near‐infrared (NIR) probes exhibit high penetration depth, low phototoxicity, and low background interference, and thereby have been used for noninvasive detection and imaging in vivo (Guo et al., 2014).

To date, several NIR fluorescent probes for β‐Gal have been developed, as reviewed previously (Yao et al., 2021; Zhang et al., 2019), but few have been applied to comprehensively detect senescence in aging and senescence‐related diseases (Liu et al., 2021). In addition, many commercial lysosomal probes are pH‐dependent, which limits their long‐term detection of lysosomal enzymes (such as β‐Gal) as they may exit the lysosomes and/or their fluorescence may be quenched once lysosomal pH increases (Zhang et al., 2014). Thus, it is imperative to develop new NIR probes to enable the precise and long‐term monitoring of SnCs in vitro and in vivo. An ideal NIR fluorescent probe for in vivo imaging of SnCs should possess a variety of desirable characteristics, including: (a) high sensitivity to lysosomal β‐Gal; (b) reasonable selectivity against potential competitive biospecies and interferents; (c) favorable fluorescence properties for the NIR fluorophore reporter; (d) reasonable cell permeability and photostability in in vitro studies; and (e) durable fluorescent response during living animal imaging and robustness in tissue handling and imaging in in vivo studies. We developed a novel NIR fluorescent probe that satisfied these requirements, showing extraordinary performance in vitro and in multiple aging/senescence‐associated disease models. This new NIR probe may facilitate our understanding of aging mechanisms and help in the development of new anti‐aging interventions.

## 2 RESULTS

### 2.1 Rational design and synthesis of NIR fluorescent probes

**FIGURE 1:**
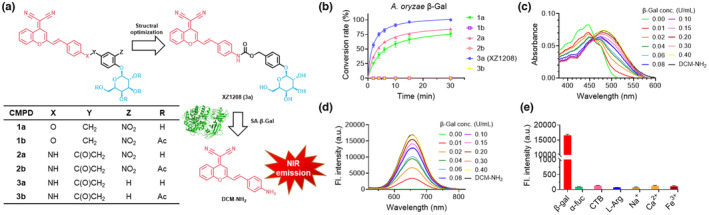
Chemical structure of NIR fluorescent probes and activation by β‐Gal. (a) Design and optimization of NIR fluorescent probes. (b) Time‐dependent cleavage of NIR fluorescent probes by β‐Gal from *A. oryzae*. (c) Characteristic absorption peak of fluorescent probe XZ1208 in presence of *A. oryzae* β‐Gal. (d) Fluorescence intensity of fluorescent probe XZ1208 after addition of *A. oryzae* β‐Gal. (e) Fluorescence emission of XZ1208 in the presence of potential competitive biospecies and interferents. Data are mean ± SEM (*n* = 3 independent assays).

Although several β‐Gal probes have been developed, their fluorophore scaffolds primarily fall within the visible region, limiting their application in in vivo imaging (Yao et al., 2021; Zhang et al., 2019). In contrast, fluorescence imaging in the NIR window (650–900 nm) offers unique advantages, such as deeper tissue penetration, lower background autofluorescence, and less biological damage (Guo et al., 2014). Here, we started with a dicyanomethylene‐4*H*‐pyran (DCM) scaffold due to its emission wavelength in the NIR region, high photostability, large Stokes shift, low cytotoxicity, pH insensitivity, and broad synthetic accessibility (Guo et al., 2012). The β‐Gal probe consisted of three moieties: that is, DCM‐derived NIR fluorophore reporter, β‐Gal residue, and self‐immolative linker (Figure 1a). The NIR fluorescence off–on switch is triggered by cleavage of the glycosidic bond, followed by transformation of the self‐immolative unit to quinone methides and eventual activation of the NIR fluorophore reporter. The synthetic route of the probe (Scheme S1) and structural characterization are described in the Supporting Information.

We first performed time‐dependent high‐performance liquid chromatography (HPLC) analysis of enzymatic hydrolysis products of the designed probes in the presence of β‐Gal from *Aspergillus oryzae*, which is a lysosomal enzyme that possesses a similar catalytic domain as human SA‐β‐Gal (Li et al., 2020). As shown in Figure 1b, the linkage between the self‐immolative moiety and fluorophore reporter affected reporter release and probe 2a, with a carbamate connection, showed a better effect than the ether connection analog 1a. Subsequent modification to remove the nitro group on the linker further improved release efficacy (3a vs. 2a), probably due to electron density adjustment on the aromatic ring accelerating the self‐immolation rate (Alouane et al., 2015). Acetylated β‐galactose has been applied in the prodrug design of senolytics (Cai et al., 2020; González‐Gualda et al., 2020). However, in our cell‐free assay, the corresponding Ac‐analogs completely lost their activity (1a vs. 1b, 2a vs. 2b, and 3a vs. 3b). A similar reactivity and structure–activity relationship was observed in the presence of bovine β‐Gal (Supporting Information). Replacing *A. oryzae* β‐Gal with *Escherichia coli* β‐Gal expressed in the cytoplasm resulted in delayed cleavage (Supporting Information), which can be attributed to compromised binding preference. In all above assays, probe 3a (XZ1208) exhibited the highest release rate of the active NIR fluorophore reporter.

Subsequently, we investigated the spectroscopic properties of the fluorescent probe XZ1208. The characteristic absorption peak of XZ1208 at 447 nm decreased with β‐Gal incubation, while a new absorption signal appeared at 484 nm (Figure 1c). Upon excitation at 484 nm, XZ1208 displayed very weak fluorescence in the absence of β‐Gal (Φ_XZ1208_ = 0.003), indicating that the intramolecular charge transfer (ICT) process was inhibited by the conversion of the DCM‐NH_2_ amino group to carbamate. In contrast, the addition of β‐Gal resulted in a significant enhancement of fluorescence at 654 nm (Figure 1d), supporting the cleavage of glycosidic bonds to liberate DCM‐NH_2_, leading to fluorescence turn‐on (Φ_DCM‐NH2_ = 0.447). The Stokes shift was as large as 170 nm, which is desirable for high‐quality optical imaging. We evaluated selectivity over potential competitive biospecies, such as alpha‐L‐fucosidase (α‐fuc) and lysosomal enzyme cathepsin B (CTB), as well as several potential interferents, including L‐arginine, Na^+^, Ca^2+^, Fe^3+^, Cl^−^, SO_3_^2−^, and NO_3_^−^, and found minimal fluorescence response (Figure 1e). Thus, given its favorable spectroscopic properties, XZ1208 is a suitable NIR fluorescent probe for biological evaluation.

### 2.2 Highly specific labeling of SnCs with fluorescent probes in vitro

**FIGURE 2:**
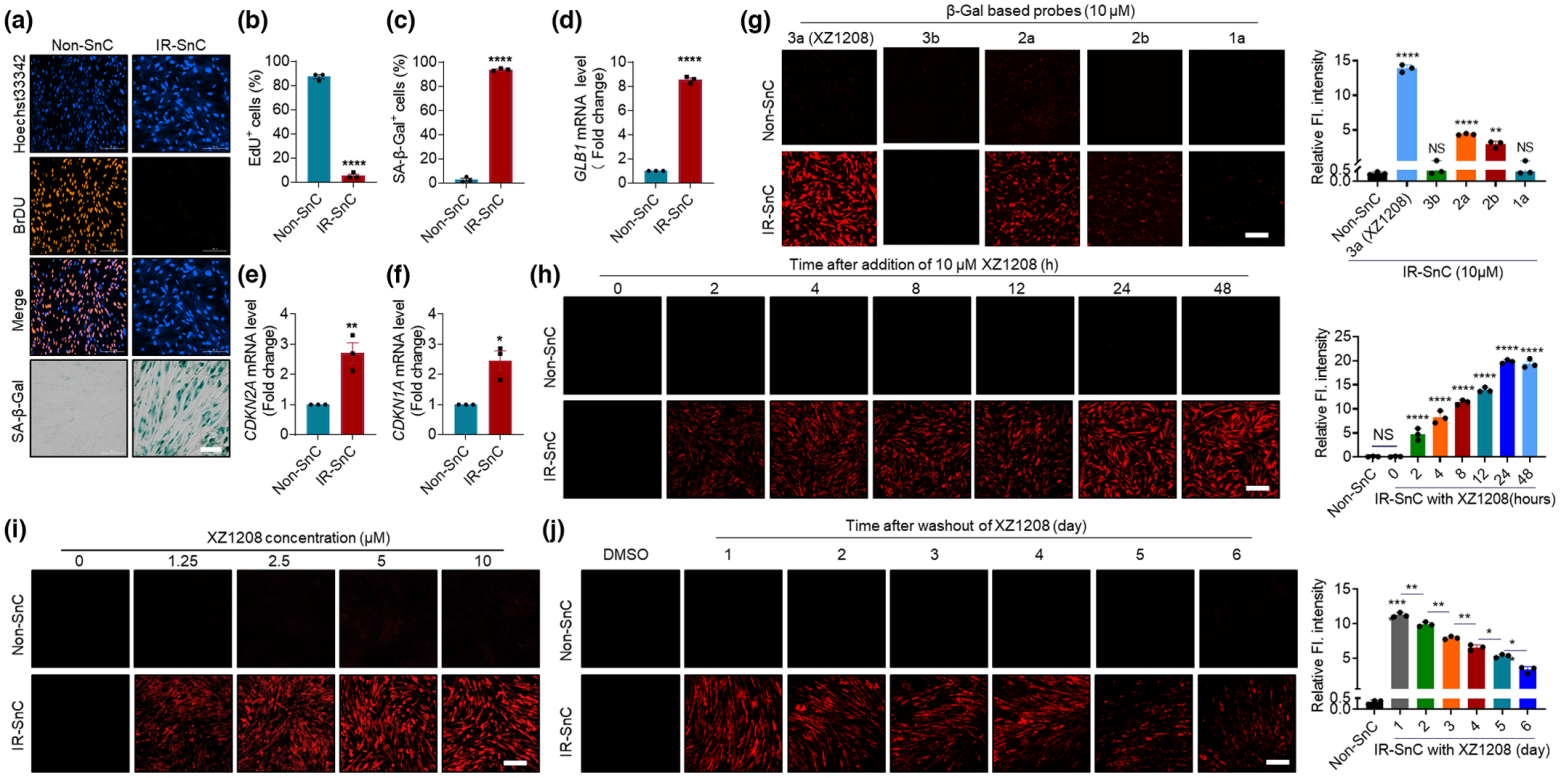
Specific activation of XZ1208 in senescent HEL fibroblasts. (a) β‐Gal staining and DNA synthesis in HEL Non‐SnCs and IR‐SnCs. (b) Quantification of EdU‐positive non‐SnCs and IR‐SnCs. (c) Quantification of SA‐β‐Gal‐positive non‐SnCs and IR‐SnCs. (d) mRNA levels of *GLB1* encoding β‐Gal in non‐SnCs and IR‐SnCs. (e, f) mRNA levels of *CDKN2A* and *CDKN1A* in non‐SnCs and IR‐SnCs. (g) Confocal imaging and fluorescence intensity quantification of non‐SCs and IR‐SnCs at 48 h after addition of indicated probes (10 μM). (h) Confocal imaging and fluorescence intensity quantification of non‐SCs and IR‐SnCs at different time points after addition of XZ1208 (10 μM). (i) Confocal imaging of non‐SnCs and IR‐SnCs at 48 h after addition of indicated concentrations of XZ1208. (j) XZ1208 (10 μM) was added to non‐SCs and IR‐SnCs for 48 h, with cells then washed and imaged by laser scanning confocal microscopy. Data are mean ± SEM (*n* = 3 independent assays). Representative images and quantification are presented. Scale bars, 200 μm (for a and j) and 100 μm (for g, h, and i). *p < 0.05, **p < 0.01, ***p < 0.001, and ****p < 0.0001 compared to non‐SnC group or indicated groups. NS, not significant.

We next tested whether the above fluorescent probes could be activated by SA‐β‐Gal in SnCs induced by x‐ray irradiation. Cellular senescence was confirmed by increased SA‐β‐Gal activity, enlarged cell and nuclear size, decreased DNA synthesis, and increased mRNA levels of *GLB1* encoding β‐Gal, *CDKN2A* (p16), *CDKN1A* (p21), and SASP factors in irradiation‐induced senescent (IR‐SnC) human embryonic lung (HEL) fibroblasts (Figure 2a–f and Figure S1a–g). Consistent with the cell‐free assays (Figure 1b–d), 3a (XZ1208) was completely cleaved to release the fluorescent dye (showing bright red), 2a was secondarily activated, while probe 1a and acetylated analogs 3b and 2b showed minimal fluorescence signals in the HEL IR‐SnCs, likely due to poor cell permeability and/or inefficient ester hydrolysis (Figure 2g, lower panel). The fluorescence signal of XZ1208 was well colocalized with that of LysoTracker (Figure S2a), suggesting that XZ1208 was cleaved and activated mainly in the lysosome. In contrast, none of the probes, including XZ1208, were activated or exhibited detectable fluorescence signals in non‐senescent cells (Non‐SnCs) (Figure 2g, upper panel). To validate these results, we tested the fluorescent probes in human dermal fibroblasts (HDF). Similar to HEL cells, irradiated HDF cells became senescent, as indicated by senescence markers (Figure S2b–n). Consistently, XZ1208 showed excellent specific labeling of HDF IR‐SnCs (Figure S2o).

Given its complete cleavage by β‐Gal in the cell‐free assays and in vitro experiments (Figures 1b and 2G; Figure S2o), we next explored the efficacy of XZ1208 in detecting senescence. XZ1208 was rapidly cleaved within 2 h and the fluorescence signal peaked at 24 h and remained stable at 48 h in HEL (Figure 2h) and HDF IR‐SnCs (Figure S2p). XZ1208 efficiently labeled SnCs, even at a low concentration of 1.25 μM (Figure 2i and Figure S2q, r). In contrast, there were no observable fluorescence signals in HEL non‐SnCs or HDF non‐SnCs (Figure 2h–j and Figure S2o–s). To test the duration of the effect of XZ1208 labeling on senescence after activation, we treated Non‐SCs and IR‐SnCs with 10 μM XZ1208 for 48 h, then imaged the cells at different time points after removal from the culture medium. Results showed that the fluorescence signal was still detectable in HEL and HDF IR‐SnCs for 6 days after washing (Figure 2j and Figure S2s), suggesting the long‐term persistence of XZ1208 labeling of SnCs. We also tested fluorescence in cells after incubation with the free fluorophore (DCM‐NH_2_) and negative control probe 3b. As shown in Figure S3, DCM‐NH_2_ showed significant signals in both HEL non‐SnCs and IR‐SnCs, while signals were not detectable in either non‐SnCs or IR‐SnCs for 3b.

To further validate the efficacy of XZ1208 in labeling SnCs, we induced senescence in HEL and HDF cells by extensive replication (REP‐SnCs). With increasing number of passages, the HEL cells showed decreased DNA synthesis (Figure S4a, b), decreased cumulative population doubling level (CPDL) (Figure S4c), increased SA‐β‐Gal activity (Figure S4a, d), and increased mRNA levels of *CDKN2A* (p16) and *CDKN1A* (p21) (Figure S4e, f). When the different passaged HEL cells were treated with XZ1208, only the p31 and p41 passaged cells showed obvious XZ1208 staining (Figure S4g, h). The same results were obtained for the HDF REP‐SnCs (Figure S5a–h).

As XZ1208 achieved long‐term labeling of SnCs (Figure 2j and Figure S2s), we next assessed whether XZ1208 causes toxicity to the cells. We treated HEL and HDF non‐SnCs and IR‐SnCs with higher doses of XZ1208 (up to 50 μM) for 3 days but did not observe any changes in cell viability (Figure S6a, b), indicating safety when used to label senescence in vitro. These findings suggest that XZ1208 exhibits considerable advantages, such as high specificity, low toxicity, and long‐term duration, in detecting β‐Gal activity in SnCs against non‐SnCs in vitro.

### 2.3 Performance of XZ1208 in labeling SnCs in senescence mouse models

**FIGURE 3:**
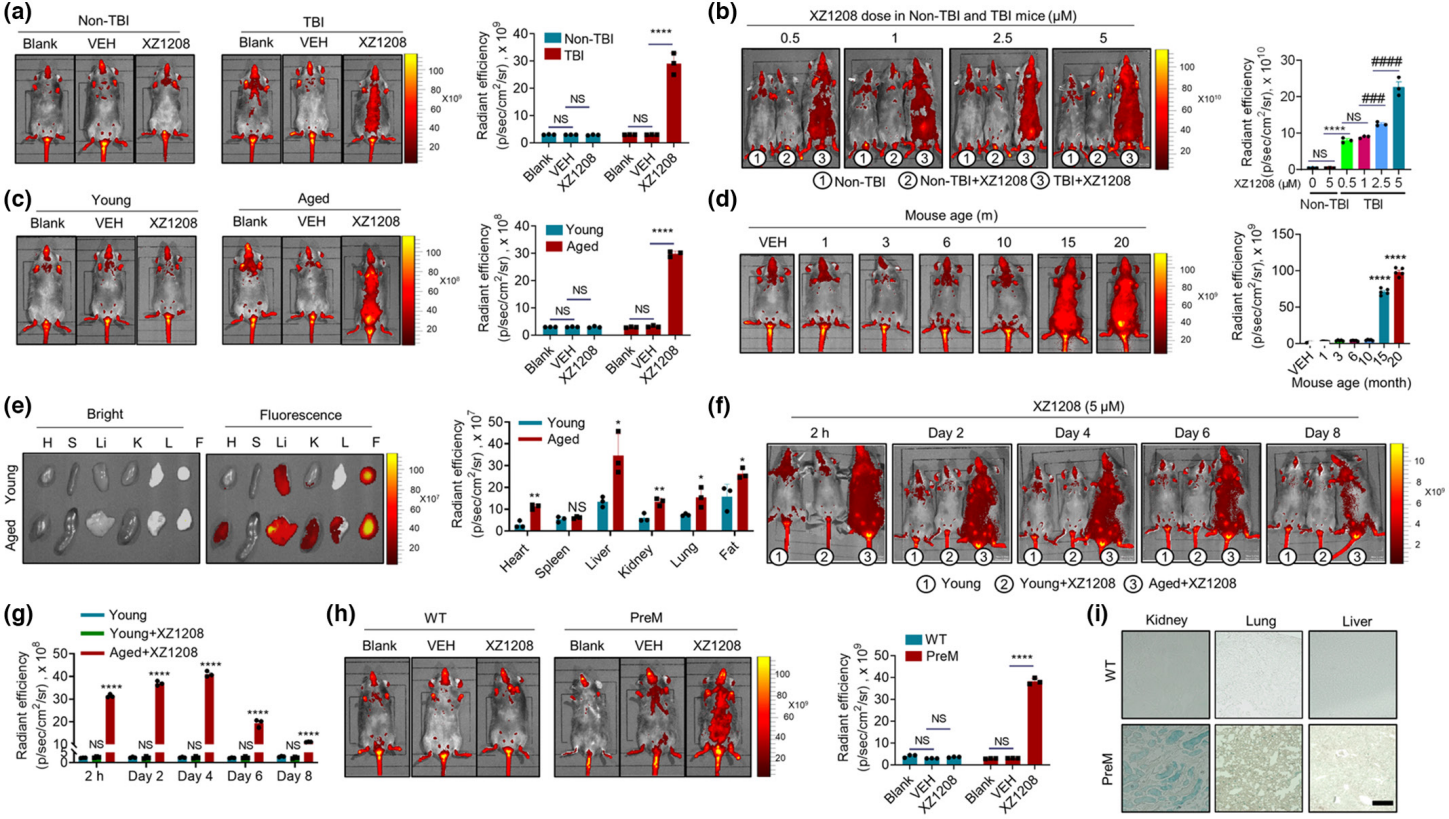
Specific and long‐term labeling by XZ1208 in senescence mouse models. (a, b) Non‐TBI and TBI mice were intravenously injected with vehicle (VEH), 5 μM XZ1208 (a), or indicated concentrations of XZ1208 (b) via the tail vein, then imaged and quantified for fluorescence intensity at 24 h after injection (*n* = 3 mice per group). (c, d) Young mice aged 2–3 months, old mice aged 20 months (*n* = 3 per group), or different aged mice were intravenously injected with vehicle (VEH), or 5 μM XZ1208 via the tail vein, then imaged and quantified for fluorescence intensity at 24 h after injection (*n* = 5 mice per group). (e) Major organs/tissues from young mice aged 2–3 months and aged mice aged 20 months were collected, imaged, and quantified for fluorescence intensity at 24 h after intravenous injection with 5 μM XZ1208 via the tail vein. H = heart, S = spleen, Li = liver, K = kidney, L = lung, F = fat. (f, g) Young mice aged 2–3 months and old mice aged 20 months were intravenously injected with 5 μM XZ1208 via the tail vein, then imaged and quantified for fluorescence intensity at indicated time points after injection (*n* = 3 per group). (h) Wild‐type (WT) mice and prematurely (PreM) aged mice were intravenously injected with 5 μM XZ1208 via the tail vein, then imaged and quantified for fluorescence intensity at 24 h after injection (*n* = 3 mice per group). (i) β‐Gal staining of kidney, lung, and liver in WT and PreM mice. Scale bar, 200 μm. *p < 0.05, **p < 0.01, ***p < 0.001, and ****p < 0.0001 compared to VEH or indicated groups. ^#^ *p* < 0.05, ^##^ *p* < 0.01, ^###^ *p* < 0.001, and ^####^ *p* < 0.0001 compared to indicated groups. NS, not significant.

Given the excellent performance of XZ1208 in detecting intracellular SA‐β‐Gal activity in vitro, we next examined its suitability for SnC imaging in mouse models of senescence. We first tested its efficiency in total body irradiated (TBI) mice (He, Zhang, et al., 2020). Three months after TBI, SnCs accumulated in mouse tissues, with increased mRNA levels of *cdkn2a* and SASP factors (He, Zhang, et al., 2020) and increased SA‐β‐Gal activity (Figure S7a). Autofluorescence was low in Non‐TBI and TBI mice (Figure 3a and Figure S7b). After free fluorophore (DCM‐NH_2_) administration, both non‐TBI and TBI mice showed increased fluorescence signals (Figure S7b). In contrast, only TBI mice showed bright fluorescence signals after injection with 5 μM XZ1208 (Figure 3a), as validated in tissue sections by confocal microscopy (Figure S8a, b). These data suggest that XZ1208 exhibits good specificity in the labeling of SnCs in TBI mice. Next, we injected mice with different concentrations of XZ1208 and found a dose‐dependent increase in fluorescence signals (Figure 3b). XZ1208 showed higher sensitivity in labeling SnCs compared to existing probes, even at low concentrations (0.5 μM), and reached a saturation point (5 μM XZ1208) for fluorescence signals (Figure 3b).

To verify its performance, we tested XZ1208 in naturally aged mice. Similar to that observed in TBI mice, the autofluorescence signal was very low, and the free fluorophore (DCM‐NH_2_) showed increased fluorescence signals in both young and 20‐month‐old mice (Figure 3c; Figure S7c). In contrast, XZ1208 showed strong fluorescence signal selectivity in aged mice (Figure 3c; Figure S7c). The ration in the fluorescence signal of XZ1208‐treated aged/young mice was greater than that of DCM‐NH_2_‐treated aged/young mice (9.73 vs. 1.18 at 60 min; 11.84 vs. 2.31 at 24 h) (Figure S7d), suggesting significantly higher selectivity of XZ1208 in labeling SnCs than DCM‐NH_2_. We next injected XZ1208 into different aged mice (1, 3, 6, 10, 15, and 20 months) to test the SnC burden. Results showed that SnCs accumulated in mouse tissues, as indicated by increased SA‐β‐Gal activity in 15‐ and 20‐month‐old mice (Figure S7e). Consistently, 15‐ and 20‐month‐old mice were strongly labeled by XZ1208 with a robust fluorescence signal, while 10‐month‐old mice showed weaker signals comparable to younger mice (Figure 3d), indicating that XZ1208 precisely labeled SnCs in naturally aged mice. These results suggest that mice were burdened with SnCs, as evidenced by the strong increase in fluorescence starting at 15 months of age (Figure 3d). We then wondered which organ/tissues were most burdened by SnCs in aged mice. After injection of 5 μM XZ1208, major organs/tissues were collected and imaged. Compared with young mice, we found more SnCs in the heart, liver, kidney, and lung of aged mice (Figure 3e). However, the highest fluorescence signals were found in the liver and fat (Figure 3e), which could be partially attributed to its hydrophobicity, but more likely to SnC accumulation in the two organs/tissues, as liver and fat are more easily to undergo senescence compared to the other organs/tissues (Yousefzadeh et al., 2020). Unexpectedly, we did not observe a noticeable signal in the spleen (Figure 3f), consistent with previous research using a NIR probe to label senescent tumor cells induced by camptothecin (Liu et al., 2021). The fluorescence intensity results were consistent with SA‐β‐Gal staining and fluorescence detection by confocal microscopy of tissue sections (Figure S8c, d), indicating strong positive signals in the kidney, lung, and liver of 20‐month‐old mice.

We next wondered how quickly XZ1208 is activated and how long the labeling effects last in vivo. We administered 20‐month‐old mice with a single dose of 5 μM XZ1208 intravenously via the tail vein, followed by imaging at different time points. XZ1208 was completely activated within 1–2 h and showed a very strong fluorescence signal (Figure 3f, g and Figure S7c). Notably, the labeling effect persisted for at least 6 days, with a slight downward trend on Day 8 (Figure 3f, g), suggesting that XZ1208 achieved long‐term detection of SnCs in aged mice. To further validate its performance, we tested XZ1208 in prematurely aged *Wrn* and *Terc* (encoding telomerase RNA component) null mice, which show accelerated replicative senescence and accumulation of DNA‐damage foci in cultured cells (Chang et al., 2004). Here, 5 μM XZ1208 effectively labeled SnCs in the prematurely aged mice compared to wild‐type mice (Figure 3h and Figure S8e, f), likely due to the higher SA‐β‐Gal activity (Figure 3i).

Considering its sustained labeling in vivo, we also wondered whether XZ1208 causes any potential toxicity. As such, we injected mice with a higher dose of XZ1208 (10 μM) and tested blood cell composition, plasma biochemical indices, and tissue histomorphometry on Day 7 after administration. We did not observe any differences in tissue histopathology (Figure S9a), blood cell counts, or percentages between the vehicle (VEH)‐ and XZ1208‐treated groups (Figure S9b). Kidney and heart function indicators were unchanged, or even showed a slight decrease (e.g., alanine transaminase (ALT), which increases under liver damage) (Figure S9c). Thus, these findings demonstrate that XZ1208 accurately reports the presence of SnCs in senescence mouse models and achieves long‐term and safe SnC labeling.

### 2.4 XZ1208 allows detection of senolytic effects of ABT263

**FIGURE 4:**
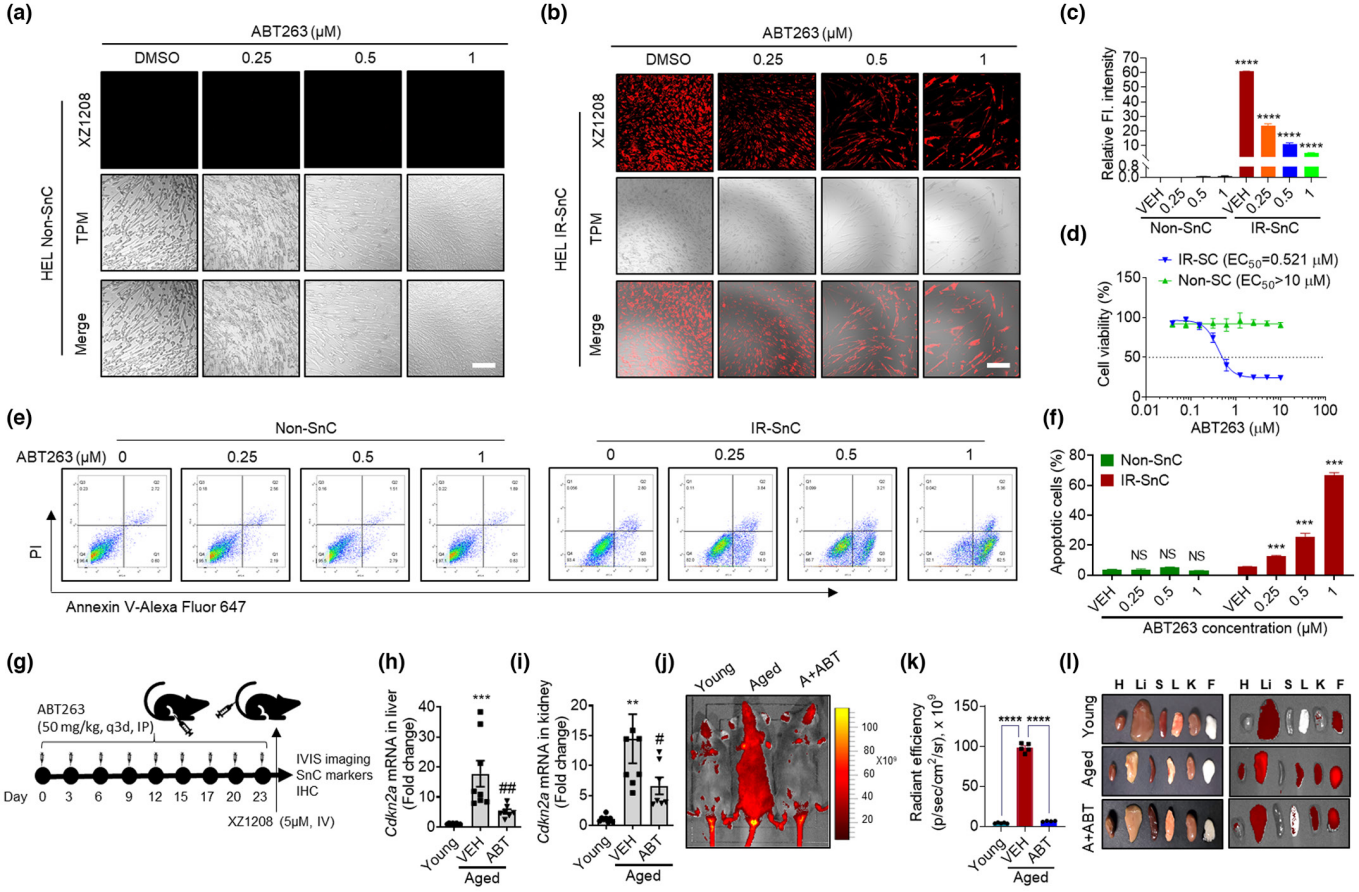
Detection of senolytic effects of ABT263 by XZ1208 in vitro and in vivo. (a, b) HEL non‐SnCs and IR‐SnCs were treated with indicated concentrations of ABT263 for 72 h, incubated with 10 μM XZ1208 for 48 h, and imaged via confocal microscopy. Scale bar, 200 μm. (c) Quantification of fluorescence intensity for (a, b). Data are mean ± SEM (*n* = 3 independent assays). (d) HEL non‐SnCs and IR‐SnCs were treated with indicated concentrations of ABT263, with viability determined by MTS assay at 72 h after ABT263 treatment. EC_50_, half‐maximal effective concentration. Data are mean ± SEM (*n* = 3 independent experiments). (e) ABT263 induced apoptosis selectively in HEL IR‐SnCs in a dose‐dependent manner. HEL non‐SnCs and IR‐SnCs were treated with indicated concentrations of ABT263 for 72 h. Representative flow cytometry analysis of apoptosis is shown. PI, propidium iodide. (f) Quantification of apoptotic cells in (e). Data are mean ± SEM (*n* = 3 independent assays). (g) Illustration of experimental design. Mice aged 20 months (aged) were given 50 mg/kg ABT263 via intraperitoneal injection every 3 days (q3d) for a total of nine injections, mice were injected with 5 μM XZ1208 intravenously via the tail vein and imaged after 24 h. After imaging, mice were euthanized to harvest various tissues for analysis. (h, i) Expression of *Cdkn2* in liver (h) and kidney (i) was measured by qRT‐PCR (*n* = 8 mice for young and aged groups, *n* = 7 mice for ABT263‐treated group). (j–l) Mice and major organs/tissues were imaged at 24 h after injection with 5 μM XZ1208 (five mice from (g) were randomly selected for XZ1208 injection and imaging). H = heart, Li = liver, S = spleen, L = lung, K = kidney, F = fat. Data are mean ± SEM. Data were analyzed by one‐way ANOVA with Tukey's *post hoc* tests. *p < 0.05, **p < 0.01, ***p < 0.001, and ****p < 0.0001 compared to vehicle (VEH) in d and f; ^#^ *p* < 0.05, ^##^ *p* < 0.01, ^###^ *p* < 0.001, and ^####^ *p* < 0.0001 compared to VEH group in H, I, and K. NS, not significant.

SnCs are implicated in aging and various age‐related diseases (Chaib et al., 2022; Childs et al., 2017; He & Sharpless, 2017). Selective elimination of SnCs via transgenic or pharmaceutical strategies can treat age‐related diseases and extend health span (Baker et al., 2016; Di Micco et al., 2021; Kirkland & Tchkonia, 2020; van Deursen, 2019). To test whether XZ1208 can detect senolytic effects, we treated non‐SnCs and SnCs with ABT263, a widely used senolytic (Chang et al., 2016). As shown in Figure 4a, b, ABT263 selectively eliminated SnCs without affecting the viability of non‐SnCs, as determined by confocal, MTS, and flow cytometry assays (Figure 4a–f). As above, XZ1208 was not cleaved in non‐SnCs, but was cleaved in IR‐SnCs (Figure 4a–c). The fluorescence signal accurately reflected density changes in SnCs, suggesting that XZ1208 can detect senolysis in vitro. Next, we treated 20‐month‐old mice with 50 mg/kg ABT263 intraperitoneally (Figure 4g) and measured major senescence markers. Results showed that aged mice had higher *cdkn2a* gene expression compared to young mice, but expression was significantly reduced by ABT263 (Figure 4h, i), consistent with representative SA‐β‐Gal staining of tissue sections (Figure S10). The aged mice also showed much stronger fluorescence signals after XZ1208 injection in the tail vein compared to young mice, with a dramatic reduction following ABT263 treatment (Figure 4j, k). We then isolated and imaged major organs from mice and found that the fluorescence signal in aged organs/tissues was reduced by ABT263 (Figure 4l). These results suggest that ABT263 can effectively eliminate SnCs in aged mice, and that XZ1208 is an ideal probe for detecting senolytic effects in vivo.

### 2.5 Imaging of XZ1208 in senescence‐associated fibrotic models

**FIGURE 5:**
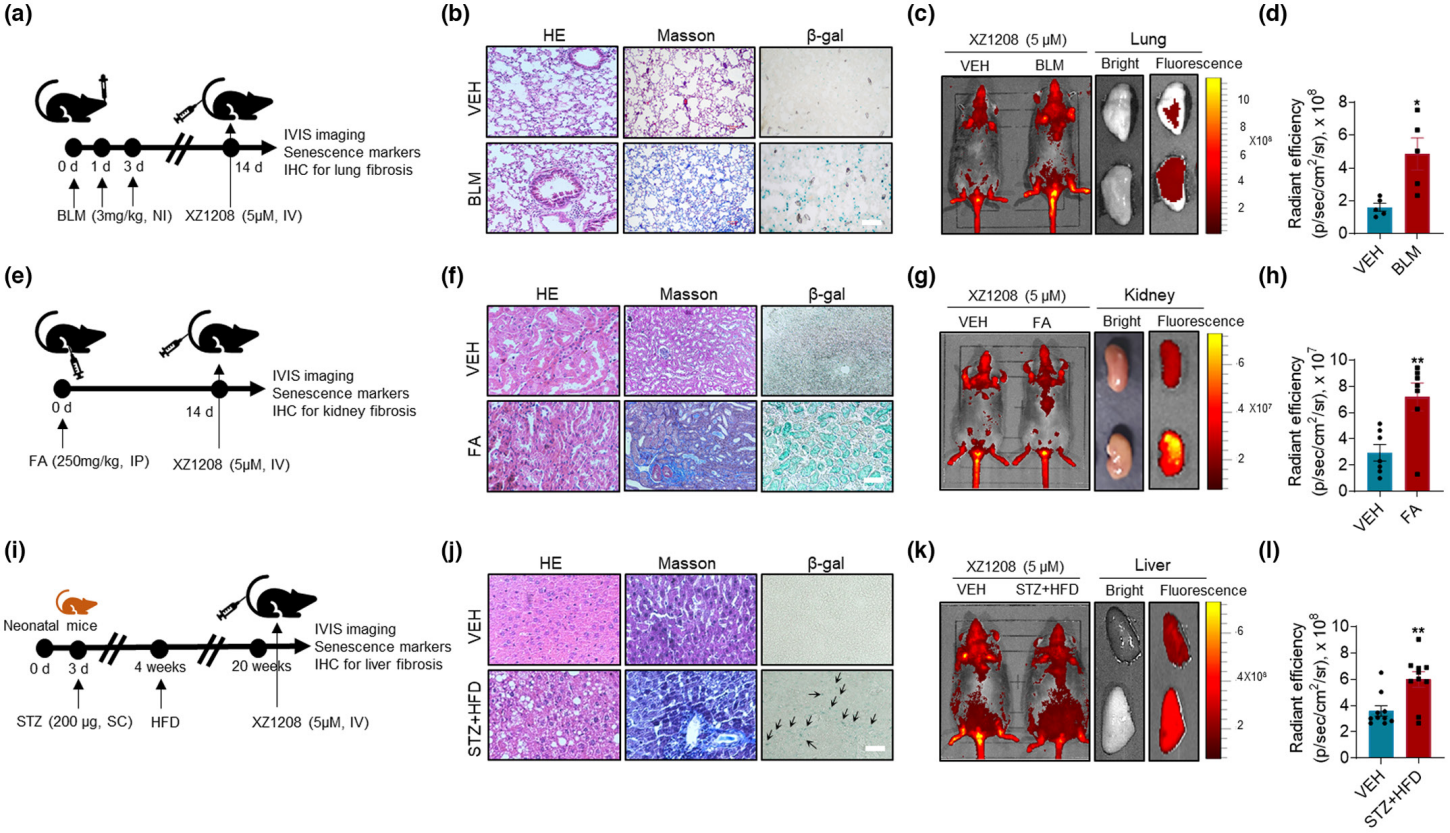
Effective labeling of SnCs by XZ1208 in fibrosis mouse models. (a–d) Lung fibrosis was induced by administering mice with 3 mg/kg BLM via nasal inhalation, followed by Masson and β‐Gal staining of the lung. Mice and lungs were imaged and quantified for fluorescence intensity (*n* = 5 mice for each group). (e–h) Kidney fibrosis was induced by administering mice with a single dose of 250 mg/kg folic acid intraperitoneally, followed by Masson and β‐Gal staining of the kidney. Mice and kidneys were imaged and quantified for fluorescence intensity (*n* = 7 mice for each group). (i–l) Liver fibrosis was induced by injecting neonatal mice subcutaneously with 200 μg of STZ, followed by a HFD. Liver fibrosis was validated by Masson staining and SnC accumulation was indicated by β‐Gal staining. Mice and livers were imaged and quantified for fluorescence intensity (*n* = 10 mice for each group I). Scale bars, 100 μm (for b) and 200 μm (for f and j). Black arrows indicate positive β‐Gal staining. Data are mean ± SEM. Data were analyzed by two‐sided Student's *t*‐test. *p < 0.05, **p < 0.01, ***p < 0.001, and ****p < 0.0001 compared to vehicle (VEH) group.

In addition to the aging process, SnCs also accumulate and participate in various fibrotic diseases, such as pulmonary, liver, and renal fibrosis. Here, we constructed these fibrosis mouse models using chemical or chemical plus diet approaches to test the accumulation of SnCs with XZ1208. As shown in Figure 5a, mice treated with bleomycin (BLM) via nasal inhalation displayed marked pulmonary fibrosis, as indicated by collagen fiber accumulation based on Masson staining (Figure 5b). An increase in SA‐β‐Gal staining‐positive cells in the tissue sections of BLM‐treated mice suggested accumulation of SnCs in the lung after treatment (Figure 5b). After injection with XZ1208, we observed a threefold increase in fluorescence signals in the lung of the fibrotic group compared to the VEH (Figure 4c, d and Figure S11a), consistent with tissue section imaging by confocal microscopy (Figure S11b).

To confirm the efficacy of XZ1208, we established a renal fibrosis mouse model by intraperitoneally injecting mice with a single dose of 250 mg/kg folic acid (FA) (Figure 5e). Similarly, the FA‐treated mice showed intense blue Masson staining, indicating renal fibrosis in the kidney (Figure 5f). Although we did not observe a significant change in fluorescence signals in mice, we did observe a 2.4‐fold increase in fluorescence signals in the kidney and tissue sections of FA‐treated mice (Figure 5g, h and Figure S11c, d), consistent with the changes in SA‐β‐Gal staining (Figure 5f).

Liver fibrosis is a wound healing process initiated in response to liver injury caused by factors such as alcohol abuse, metabolic disorders, viral infection, and drug use (Udomsinprasert et al., 2021). In recent years, cellular senescence has been shown to have diagnostic, prognostic, and therapeutic potential in liver fibrosis (Udomsinprasert et al., 2021). Here, we constructed a liver fibrosis mouse model using streptozotocin (STZ) in combination with a high‐fat diet (HFD) (Figure 5i) (Fujii et al., 2013). The STZ + HFD‐challenged mice exhibited severe liver fibrosis accompanied by SnC accumulation in the liver (Figure 5j). Consistently, the STZ + HFD‐treated mice showed a stronger fluorescence signal in the liver and tissue sections after injection with XZ1208 (Figure 5k, l and Figure S11e, f). These data suggest that XZ1208 can effectively label SnCs in various fibrotic disease models.

### 2.6 Application of XZ1208 in wound healing model

**FIGURE 6:**
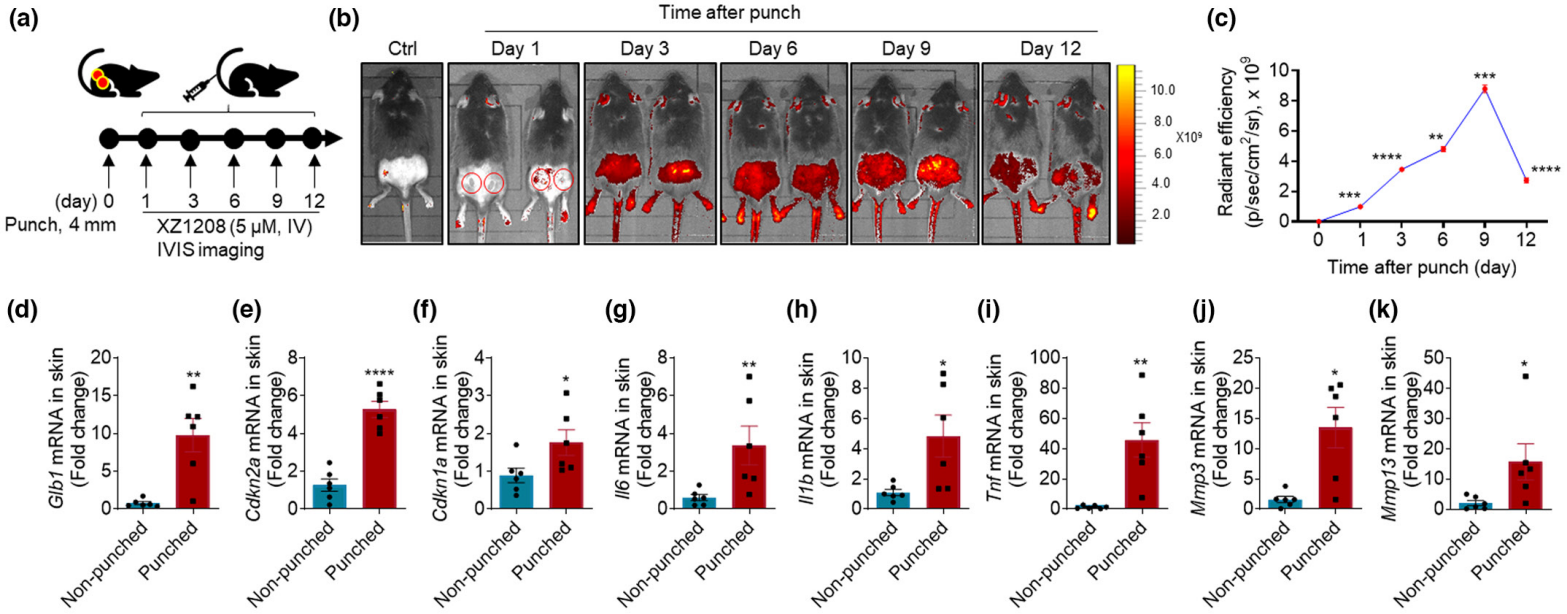
Indication of SnC kinetics by XZ1208 in wound healing models. (a, b) Dorsal skin of mice was wounded using 4‐mm punches, then imaged using the IVIS imaging system at indicated time points after intravenous injection with 5 μM XZ1208 via the tail vein (*n* = 3 for each time point). Red solid or hollow circles indicate punch sites. (c) Quantification of fluorescence signals in (b). (d–k) mRNA levels of *Glb1*, *Cdkn2a*, *Cdkn1a*, and SASP factors in skin on Day 6 after punch (*n* = 5 mice for non‐punched and punched groups, respectively). Data were analyzed by two‐sided Student's *t*‐test or Mann Whitney test. *p < 0.05, **p < 0.01, ***p < 0.001, and ****p < 0.0001 compared to non‐punched group (Day 0).

As SnCs arise in response to cutaneous wounds and play an essential role in cutaneous wound healing (Demaria et al., 2014), we used XZ1208 to assess the dynamic accumulation of SnCs in a skin injury model. In brief, mice received 4‐mm full‐thickness punch biopsy wounds to the dorsal flanks (Figure 6a). As indicated by the fluorescence signal after XZ1208 activation, a small number of SnCs was detected on Day 1 after wounding, but a significant number were apparent on Day 3 (Figure 6b, c); the fluorescence signal sharply increased on Day 6 after wounding, peaked on Day 9, then declined slightly on Day 12 (Figure 6b, c). These results are similar to previous research, in which 6‐mm punch‐injured p16‐3MR mice showed a peak in SnC accumulation on Day 6 after wounding (Demaria et al., 2014). To confirm our results, we collected skin tissue samples on Day 6 after wounding and tested for classical senescence markers. As shown in Figure 6d, the mRNA level of *Glb1* encoding β‐Gal was more than nine times higher in the punched group than in the non‐punched group. Consistently, the punched mice showed significantly higher mRNA levels of *cdkn2a* (Figure 6e), *cdkn1a* (Figure 6f), and SASP factors, including *Il6*, *Il1b*, *Tnf*, *Mmp3*, and *Mmp13* (Figure 6g–k), and higher fluorescence signals (Figure S12) in the skin, indicating abundant SnC accumulation after cutaneous wounding. These results suggest that XZ1208 can well report the kinetics of SnCs in wound healing models.

## 3 DISCUSSION

In this study, we developed a sensitive NIR probe (XZ1208) for the detection of β‐Gal in SnCs in vitro and in vivo. The probe showed almost no activation in non‐SnCs or young mice but full activation by β‐Gal in SnCs and aged mice, suggesting high specificity in labeling and differentiating SnCs from non‐SCs. In particular, XZ1208 exhibited various advantages, including non‐toxicity, good cell permeability, and turn‐on fluorescence properties, making it a powerful fluorescent tool for tracking SnCs. Compared to existing NIR probes, XZ1208 displayed a series of improvements, including good performance in testing SA‐β‐Gal, long‐term labeling of SnCs, and accurate identification of the anti‐aging effects of senolytics in vivo.

As the central hallmark of aging, cellular senescence is associated with and plays a critical role in almost all other hallmarks of aging (Ge et al., 2021; Hernandez‐Segura et al., 2018; Hu et al., 2022). Accumulating evidence indicates that the accumulation of SnCs in organs is causally implicated in individual aging and various age‐related diseases (Baker et al., 2016; Childs et al., 2017). Accordingly, clearance of SnCs has emerged as a therapeutic approach to treat such diseases (Childs et al., 2017; Di Micco et al., 2021). Exploring the underlying mechanisms of cellular senescence is a prerequisite for designing precise therapeutic strategies to target SnCs and further treat age‐related diseases (Hu et al., 2022; Zhu et al., 2015). We and others have reported the development of senolytics that can selectively kill SnCs without causing significant toxicity to normal cells (Ge et al., 2021; He, Li, et al., 2020; Hu et al., 2022; Kirkland & Tchkonia, 2020; van Deursen, 2019). However, to determine the effects of senolytic or other anti‐senescence agents, we need a precise method to detect changes in SnCs after treatment. Several senescence markers, such as p16, p21, and SASP, are commonly adopted to evaluate anti‐senescence by examining mRNA or protein levels in treated cells or mouse tissues (Hernandez‐Segura et al., 2018). The p16‐3MR transgenic mouse model was generated to visualize and eliminate SnCs in living animals (Demaria et al., 2014). These mice carry a trimodal reporter protein (3MR) under the control of the cyclin‐dependent kinase inhibitor 2a (p16) promoter. The 3MR‐encoding transgene encodes a fusion protein consisting of *Renilla* luciferase, monomeric red fluorescent protein, and herpes simplex virus thymidine kinase, which enables the visualization and elimination of p16‐positive SnCs in vivo (Chang et al., 2016; Demaria et al., 2014). However, not all cells undergo p16‐dependent senescence. For example, in our previous study, bone marrow osteoblast progenitors showed p21‐dependent senescence (He, Zhang, et al., 2020). Likewise, the upregulation of various SASP components depends on cell type (Coppé et al., 2008). Gene expression in SnCs is also temporally dynamic and highly heterogeneous (Hernandez‐Segura et al., 2017). SA‐β‐Gal is a relatively well‐characterized senescence marker widely used to detect SnCs (Debacq‐Chainiaux et al., 2009). β‐Gal is a typical glycoside hydrolase enzyme and is often used as a reporter enzyme to explore gene expression and regulation. Recently, Sun et al. (2022) generated a Glb1‐2A‐mCherry (GAC) reporter allele at the *Glb1* gene encoding lysosomal β‐Gal and used the reporter to successfully monitor systemic aging and predict lifespan in mice. However, transgenic approaches are still limited in early diagnosis, prevention, and treatment of aging‐related pathologies. As reviewed previously, several methods have been developed to evaluate β‐Gal activity, including colorimetry, electrochemistry, PET, and MRI (Yao et al., 2021). However, most of these methods require fixing cells or sacrificing animals and are not suitable for in vivo imaging. In contrast, fluorescence probes can visualize dynamic biological processes in vitro and in vivo due to their high sensitivity and resolution.

In this study, we designed several β‐Gal‐catalyzed fluorescent probes, and finally screened XZ1208 as the lead probe. XZ1208 is activated by β‐Gal, which switches fluorescence from the “off” to “on” state, and showed good performance in distinguishing SnCs from non‐SnCs. Due to the strong signal after β‐Gal activation, XZ1208 can overcome the obstacle of intrinsic autofluorescence background in living tissues, thus enabling accurate labeling of SnCs in animals. XZ1208 is also characterized by rapid activation (within 2 h), long‐term labeling (over 1 week), and low toxicity. Thus, XZ1208 is suitable for performing real‐time, high‐resolution imaging in vivo and for studying senescence without terminating the experiments, thereby enabling long‐term study of age‐related disease progression. Moreover, the stable fluorescence signal allows the evaluation of intervention responses, such as senolytic treatment in this study. Based on the effective catalytic site, the design of β‐Gal‐targeted prodrugs to selectively kill SnCs by linking a toxic molecule to the catalytic site may be a promising approach (Cai et al., 2020; González‐Gualda et al., 2020). Although β‐Gal is a commonly used biomarker of cellular senescence, it is not completely specific for SnCs. β‐Gal activity is elevated in certain cells, such as activated macrophages and tumor cells. Macrophages have been reported to accumulate and play a harmful role in injured and aged tissues by causing chronic inflammation (Oishi & Manabe, 2016). Some cancer cells, for example, primary ovarian and colorectal cancer cells, as well as precancerous lesions also show a high level of β‐Gal activity (Valieva et al., 2022), making it difficult to distinguish β‐Gal in SnCs from that in macrophages and tumor cells. Therefore, introducing additional senescence markers, such as p16, p21, and SASP, or additional dimensions, such as lysosomal pH (Gao et al., 2021), to the fluorescent probe could help distinguish SA‐β‐Gal in SnCs from β‐Gal in other cells. In addition, as the catalytic domain of human SA‐β‐Gal expressed from *GLB1* is very different from that of bacterial β‐Gal encoded by lacZ, it will be important to develop specific probes for the detection of human β‐Gal activity to avoid false positive signals in the future (Li et al., 2020). Furthermore, some NIR probes with similar scaffolds and responsiveness have been reported, mostly for β‐Gal in tumors. They may be useful for senescence detection, but their applicability remains to be evaluated.

In conclusion, we designed and identified a new NIR probe (XZ1208) with high specificity and sensitivity for labeling SnCs in vitro and in vivo. The efficacy of XZ1208 in detecting β‐Gal was validated in multiple senescence‐associated disease models. XZ1208 shows great potential in the study of aging mechanisms and early diagnosis and treatment of age‐related diseases.

## 4 MATERIALS AND METHODS

### 4.1 Chemical synthesis

The synthesis and characterization of fluorescent probes are presented in the Supporting Information.

### 4.2 Time‐dependent HPLC analysis of probe activation by β‐Gal

HPLC spectra were determined using an Agilent LC/MSD system with a Waters ACQUITY UPLC BEH C18 column (1.7 μm, 50 × 2.1 mm) at 40°C. Gradient elution was used for HPLC with a mobile phase of acetonitrile (ACN) and water containing 0.1% formic acid. The *A. oryzae* β‐Gal (G5160), *E. coli* β‐Gal (G5635), and bovine β‐Gal (G1875) were purchased from Sigma‐Aldrich (St. Louis, MO, USA). The corresponding β‐Gal (1.2 units in 5 μL of β‐Gal from *A. oryzae* and *E. coli*; 0.6 units in 5 μL of bovine β‐Gal) and probe solution (0.5 μL, 10 mM in dimethyl sulfoxide [DMSO]) were added to phosphate‐buffered saline (PBS) buffer (994.5 μL, with 30% DMSO, v/v, pH = 4.5 for *A. oryzae* and bovine β‐Gal; pH = 7.4 for *E. coli* β‐Gal). The mixture was transferred into sample vials (150 μL each) and incubated for indicated time periods at 37°C. The resulting mixture was then quenched with ACN (350 μL). The supernatant was obtained by centrifugation at 5600 *g* for 15 min, then injected into the HPLC system for analysis. The NIR fluorophore reporter release was monitored using UV–Vis absorbance at 360 nm.

### 4.3 UV–Vis and fluorescence spectroscopy

To PBS buffer (995 μL, with 30% DMSO, v/v, pH = 4.5) containing 5 μM XZ1208 was added 5 μL of *A. oryzae* β‐Gal at various concentrations (0.01–0.4 U/mL). After incubation at 37°C for 30 min, the reaction solution was quenched with ACN (1.65 mL) and transferred to quartz cuvettes to measure absorbance. UV–Vis absorption spectra were obtained using a Shimadzu UV‐2600 spectrophotometer. The absorbance spectrum scan ranged from 380 to 600 nm (1‐nm increment). The above samples were diluted five times with ACN and transferred to quartz cuvettes to measure fluorescence. The maximum absorption wavelength (484 nm) was used to acquire emission spectra. Fluorescence spectra were recorded on a Techcomp FL970 fluorescence spectrometer.

### 4.4 Selectivity study

To determine the selectivity of β‐Gal over potential competitive biospecies and interferents, fluorescence emissions of XZ1208 solutions (5.0 μM) at 654 nm were measured in the presence of *A. oryzae* β‐Gal (0.4 U/mL), α‐L‐fucosidase (2 μg/mL, α‐fuc, HY‐P75782, MedChemExpress, Shanghai, China), cathepsin B (0.4 U/mL, CTB, C6286, Sigma‐Aldrich, St. Louis, MO, USA), cations (750 μM), anions (750 μM), and L‐arginine (750 μM) at 37°C after 1 h (λ_ex_ = 484 nm). The above samples were diluted five times with ACN and transferred to quartz cuvettes to measure fluorescence. The references of the interferents used were Na_2_SO_3_, CaCl_2_, and Fe(NO_3_)_3_, purchased from Sinopharm (Beijing, China).

### 4.5 Cell culture

The HDF cells were isolated from circumcised foreskins of healthy human donors aged 5–20 years (Xiao et al., 2022). The HEL fibroblasts were purchased from the Kunming Cell Bank of Type Culture Collection (Kunming, China). All cells were cultured in complete Dulbecco's modified Eagle medium (DMEM, C11995500BT, Gibco, MD, USA) supplemented with 10% fetal bovine serum (FBS, 35‐076‐CV, Gibco; RY‐F22, Royacel Biotechnology Co., Ltd., Lanzhou, China) and 1% penicillin/streptomycin (15140‐122, Gibco) in a humidified incubator at 37°C and 5% CO_2_.

### 4.6 Senescent cell induction

Two different methods were used for SnC induction, including ionizing radiation and extensive replication, as described previously (He, Zhang, et al., 2020). Briefly, low‐passage HDF and HEL cells (<25 passages) were used as non‐SnCs or for senescence induction. To induce SnCs by ionizing radiation (IR), HDF and HEL cells at 70% confluence were irradiated with 15 Gy using the small animal radiation research platform (Xstrahl Inc., Camberley, UK). Three days after irradiation, cells were passaged once at a 1:3 dilution. The HDF and HEL cells became fully senescent 10 days after irradiation. To induce REP‐SnCs, the HEL and HDF cells were subcultured until they ceased dividing and displayed permanent growth arrest or senescence after approximately 40 passages.

### 4.7 Live cell fluorescence imaging and quantification

Probes were dissolved in DMSO to obtain 10 mM stock solutions. HEL and HDF non‐SnCs and IR‐SnCs were seeded onto 24‐well plates and incubated with the probes at indicated concentrations up to 10 μM (containing 0.1% DMSO) at 37°C for 48 h or incubated with 10 μM XZ1208 at 37°C for indicated time points. For the washout experiment, cells were incubated with 10 μM XZ1208 at 37°C for 48 h. The culture medium was then changed to probe‐free DMEM to wash out the probes at indicated times. Cells were washed three times with PBS solution before imaging. Fluorescence imaging was recorded on a confocal laser scanning microscope (Carl Zeiss LSM 880, Germany) at 595 nm excitation and 650–701 nm emission filter settings. Quantification of images obtained from cells was performed using ImageJ software. The regions of interest (ROIs) were determined by adjusting the intensity threshold, and positive signals were measured and presented as signal intensities. For colocalization imaging of XZ1208 with LysoTracker, HEL IR‐SnCs were incubated with XZ1208 (10 μM) at 37°C for 48 h, co‐stained with LysoTracker (2.5 μM; KTC4200, Abbkine Scientific Co., Ltd., Wuhan, China) for 60 min, then stained with DAPI for 5 min. Images were taken using a cell‐imaging multi‐mode plate reader (Cytation 5, BioTek, USA). All parameters were kept consistent for each experiment during data analyses.

### 4.8 Cell viability and DNA synthesis assays

HEL and HDF non‐SnCs and IR‐SnCs were seeded onto 96‐well plates and incubated with indicated doses of XZ1208 up to 50 μM (containing 0.5% DMSO) at 37°C for 72 h. Cell viability was measured using a CellTiter‐Glo Luminescent Cell Viability Assay Kit (G1111, Promega, Madison, WI, USA) following the manufacturer's instructions. Dose–response curves were generated, and half‐maximal effective concentrations (EC50) were calculated using GraphPad software 9.0.2 (San Diego, CA, USA). DNA synthesis was measured using a Cell‐Light EdU Apollo567 In Vitro Kit (C10310‐1, Ribobio, Guangzhou, China) following the manufacturer's protocols, as described previously (Xiao et al., 2022).

### 4.9 Cell apoptosis assay

The cell apoptosis assay was performed as described previously (He, Zhang, et al., 2020). Briefly, HEL non‐SnCs and IR‐SnCs were treated with vehicle (VEH) or indicated concentrations of ABT263 at 37°C for 72 h and harvested in polystyrene round‐bottom tubes (352058, Falcon, Corning, NY, USA). The cells were stained with Alexa Fluor 647‐Annexin V (1:60, 640912, BioLegend, San Diego, CA, USA) and propidium iodide (PI, 1:60, 00‐6990, eBioscience, CA, USA) at room temperature for 30 min. Apoptotic cells were analyzed using flow cytometry (LSR Fortessa, Becton Dickinson, CA, USA).

### 4.10 Cumulative population doubling level (CPDL) analysis

For CPDL analysis, the cells at each passage were counted and passaged at the same cell density. The CPDL was calculated using the equation: PDL_new_ = PDL_old_ + log_2_(NH/NI), where NH is the harvest cell number, NI is the plating cell number, PDL_old_ is the PDL at seeding, and PDL_new_ is the PDL at counting. HEL cells at Passage 21 and HDF cells at Passage 24 were considered PDL = 0.

### 4.11 Quantitative real‐time polymerase chain reaction (qRT‐PCR)

Total RNA was extracted using TRIzol reagent (15596018, Invitrogen, CA, USA) and reverse transcribed into cDNA using a RevertAid First‐Strand cDNA Synthesis Kit (K1622, Thermo Fisher Scientific, MA, USA) according to the manufacturer's protocols. We performed qRT‐PCR using 2 × Tsingke® Master qPCR Mix (TSE201, Tsingke, Beijing, China), with actin as the endogenous normalization control. Gene expression levels were calculated using the 2^−ΔΔCt^ method. Primers used in the study are shown in Table S1.

### 4.12 SA‐β‐Gal staining

Cell SA‐β‐Gal staining was performed using a Senescence Cells Histochemical Staining Kit (CS0030, Sigma‐Aldrich) according to the manufacturer's instructions. Briefly, cells were washed with PBS and fixed at room temperature for 7 min, then incubated in staining working solution overnight at 37°C. Cells were visualized using the cell‐imaging multi‐mode plate reader Cytation 5 developed by BioTek (Winooski, VT, USA). Tissue SA‐β‐Gal staining was performed using a Senescence β‐galactosidase Staining Kit (C0602, Beyotime Biotech, Shanghai, China) according to the manufacturer's protocols. Tissues were visualized using Axio Observer3 with the Airyscan platform (Carl Zeiss Meditec AG, Jena, Germany).

### 4.13 Mice, treatments and imaging

#### 4.13.1 TBI‐induced senescence mouse model

C57BL/6 mice (2–3 months old) were obtained from the Animal Center of the Kunming Institute of Zoology (Kunming, China). Mice were irradiated with 5 Gy using the Small Animal Radiation Research Platform (Xstrahl Inc, Camberley, UK). Three months after irradiation, mice were used to evaluate SnC accumulation and fluorescence probe efficacy, as described previously (He, Zhang, et al., 2020).

#### 4.13.2 Naturally aged mice

Female C57BL/6 mice aged 1, 3, 6, 10, 15, and 20 months were purchased from Jiangsu Wukong Biotechnology Co., Ltd (Suzhou, Jiangsu, China). For senolytic treatment, 20‐month‐old female mice were randomly assigned to one of the treatment groups and intraperitoneally injected with VEH (0.2 mL/mouse, q3d, nine injections) or ABT263 (50 mg/kg/q3d, nine injections). ABT263 (923564‐51‐6, GlpBio Technology, CA, USA) was formulated in 10% DMSO, 40% polyethylene glycol 300 (25322‐68‐3, MedChemExpress, Shanghai, China), 5% polysorbate 80 (9005‐65‐6, MedChemExpress), and 45% saline. Untreated young mice (2 months old, *n* = 8 mice) were included as a control group. Various tissues were harvested for analysis after the mice were euthanized using Zoletil 50 (5 mg/kg, Virbac, Carros, France) and cervical dislocation 2 days after receiving the last injection.

#### 4.13.3 Prematurely aged mice

Prematurely aged mice (*mTer* ^*−/–*^ *Wrn* ^*−/−*^) were bred and maintained in the Animal Center of the Kunming University of Science and Technology (KUST) Medical School. The mice were inbred generation by generation and G2, G3, G4, and G5 mice were obtained. Male G3 mice (6 months old) were used to evaluate SnC accumulation and fluorescence probe efficacy.

#### 4.13.4 Fibrosis models

C57BL/6 mice (2–3 months old) were obtained from the Animal Center of the Kunming Institute of Zoology (Kunming, China) to establish kidney and liver fibrosis models. To induce renal fibrosis, mice were administered FA (250 mg/kg) in vehicle (0.2 mL of 0.3 M NaHCO_3_) or vehicle only by intraperitoneal injection. At this dose, FA induces severe nephrotoxicity and renal fibrosis 14 days after administration (Long et al., 2001). To induce liver fibrosis, mice were injected with a single subcutaneous injection of 200 μg of STZ (S0130, Sigma) 2 days after birth. Four weeks later, mice were fed a HFD for 16 weeks to induce liver fibrosis (Fujii et al., 2013). Male C57BL/6 mice (2–3 months old) were obtained from the Kunming University of Science and Technology (Kunming, China) to establish a lung fibrosis model. In brief, mice were administered BLM via nasal inhalation three times over a week.

#### 4.13.5 Skin wound healing mouse model

Two equal‐sized wounds were created on the dorsal skin of mice using a 4‐mm punch.

#### 4.13.6 Imaging

Mice were injected with 5 μM or indicated concentrations of XZ1208, probe 3b, or free fluorophore (DCM‐NH_2_) intravenously via the tail vein. Compounds were dissolved in DMSO to obtain 10 mM stock solutions. Each mouse was injected with 5 μM compound formulated in 100 μL of solution containing 1% DMSO (1 μL of 10 mM stock solution), 2% TWEEN‐80 (2 μL), and 97% saline (97 μL). DCM‐NH_2_ fluorophore was injected with the same vehicle as XZ1280 probe. For dose–response experiments, mice were injected with different concentrations of XZ1208 (0.5, 1, 2.5 and 5 μM) formulated in 100 μL of solution containing different volumes of DMSO (0.125, 0.25, 0.5, and 1 μL of 10 mM stock solution, respectively), 2% TWEEN‐80 (2 μL), and different volumes of saline (97.875, 97.75, 97.5, and 97 μL, respectively). Whole body and organ/tissue optical images were taken after 24 h or at indicated time points using the IVIS Lumina XR (Caliper Life Sciences, Waltham, MA, USA) or IVIS Spectrum System (Perkin‐Elmer, Waltham, MA, USA). Quantification of images obtained from animals was performed using Living Image software 4.2 (Caliper Life Sciences, USA). The ROIs were determined by adjusting the area of interest on mice or tissue images by choosing a square shape, and positive signals were measured and presented as signal intensities. All parameters were kept consistent for each experiment during data analyses. All experimental protocols were approved by the Animal Ethics Committee of the Kunming Institute of Zoology, Chinese Academy of Sciences, and by the Experimental Animal Ethics Committee of Kunming University of KUST Medical School. All procedures conformed to the principles of animal protection, welfare, and ethics and relevant national guidelines.

### 4.14 Hematoxylin and eosin (H&E) staining

Hematoxylin eosin staining solution (Harris) (BA4025, Baso Diagnostics, Zhuhai, China) was used to perform H&E staining according to the manufacturer's protocols. Tissues were visualized using an Axio Observer (Carl Zeiss Meditec AG, Jena, Germany).

### 4.15 Masson trichrome staining

Formalin‐fixed tissues were embedded in paraffin and sectioned at 5 μm for Masson trichrome staining (BA4079, Baso Diagnostics) following the manufacturer's protocols. Tissues were visualized using an inverted microscope (Eclipse Ti‐S, Nikon, Japan).

### 4.16 Statistical analysis

All statistical analyses were performed and figures were drawn using GraphPad Prism v9. All data are presented as means ± standard error of the mean (SEM). Comparisons were made using two‐tailed Student's *t*‐test when comparing two experimental groups. For Student's *t*‐tests that failed the normality test, the Mann Whitney test was used. For comparisons between more than two groups, one‐way analysis of variance (ANOVA) with Tukey's or Dunnett's *post hoc* test was used. *p* < 0.05 was considered significant.

## AUTHOR CONTRIBUTIONS

Conceptualization, Y.H. and X.Z.; Writing—original draft preparation, Y.H. and X.Z.; Chemical synthesis and cell‐free assays: C.D., Z.W., S.H., Y.Y., and C.C.; In vitro and in vivo experiments: L.H., M.Z., H.L., Y.Z., R.Z., S.J., and J.L. All authors reviewed and approved the manuscript.

## CONFLICT OF INTEREST STATEMENT

Y.H., X.Z., C.D., L.H., Z.W., and S.H. are inventors of a pending patent application for the development of near‐infrared probes for detecting senescent cells.

## Supporting information


Supporting Information File S1.

Supporting Information File S2.

## Data Availability

The data that support the findings of this study are available from the corresponding author upon reasonable request.
