# Supplementary material for: A rationally designed fluorescence probe achieves highly specific and long‐term detection of senescence in vitro and in vivo
Source: Aging Cell. 2023 Jun 13;22(8):e13896. doi: 10.1111/acel.13896 (PMC10410003; doi:10.1111/acel.13896)
Supplement: Supplementary file 1 — Supporting Information File S1. [file ACEL-22-e13896-s002.docx]

**Supplementary figures and table**

**A rationally designed fluorescence probe achieves highly specific and long-term detection of senescence *in vitro* and *in vivo***

Li Hu^1,2,3#^, Chanjuan Dong^4#^, Zhe Wang^5^, Shengyuan He^4^, Yiwen Yang^5^, Meiting Zi^1,2^, Huiqin Li^1,2^, Yanghuan Zhang^6^, Chuanjie Chen^3,4^, Runzi Zheng^6^, Shuting Jia^6^，Jing Liu^6^, Xuan Zhang^3,4,5*^, Yonghan He^1,2,3*^

^1^Key Laboratory of Healthy Aging Research of Yunnan Province, Kunming Institute of Zoology, Chinese Academy of Sciences, Kunming, China

^2^State Key Laboratory of Genetic Resources and Evolution, Kunming Institute of Zoology, Chinese Academy of Sciences, Kunming, China

^3^University of Chinese Academy of Sciences, Beijing, China

^4^Drug Discovery & Development Center, Shanghai Institute of Materia Medica, Chinese Academy of Sciences, Shanghai, China

^5^School of Chinese Materia Medica, Nanjing University of Chinese Medicine, Nanjing, China

^6^Laboratory of Molecular Genetics of Aging and Tumor, Medical School, Kunming University of Science and Technology, Kunming, China

* **Corresponding authors**:

Yonghan He

E-mail: heyonghan@mail.kiz.ac.cn; Tel./Fax: +86-871-65118976

Xuan Zhang

E-mail: zhangxuan@simm.ac.cn, Tel: +86-21-68077845

| **Supplementary Item** | **Title** |
| --- | --- |
| Supplementary Fig. 1 | mRNA levels of SASP factors in HEL Non-SnCs and IR-SnCs. |
| Supplementary Fig. 2 | Senescence markers and activation of fluorescence probes in IR-SnCs. |
| Supplementary Fig. 3 | Confocal images of HEL Non-SnCs and IR-SnCs after incubation with β-Gal probe XZ1208, free fluorophore DCM-NH2, or negative control probe 3b. |
| Supplementary Fig. 4 | Activation of XZ1208 in replicative senescent HEL fibroblasts. |
| Supplementary Fig. 5 | Activation of XZ1208 in replicative senescent HDF cells. |
| Supplementary Fig. 6 | Effects of XZ1208 on cell viability of Non-SnCs and IR-SnCs. |
| Supplementary Fig. 7 | β-Gal staining and imaging in mice. |
| Supplementary Fig. 8 | Confocal images of major tissue sections of mice. |
| Supplementary Fig. 9 | H&E staining of major tissues and blood parameters in mice. |
| Supplementary Fig. 10 | β-Gal staining of major tissues in mice treated with ABT263. |
| Supplementary Fig. 11 | Images of lung, kidney, and liver in fibrosis mouse models. |
| Supplementary Fig. 12 | Confocal images of skin in wound healing models. |

**
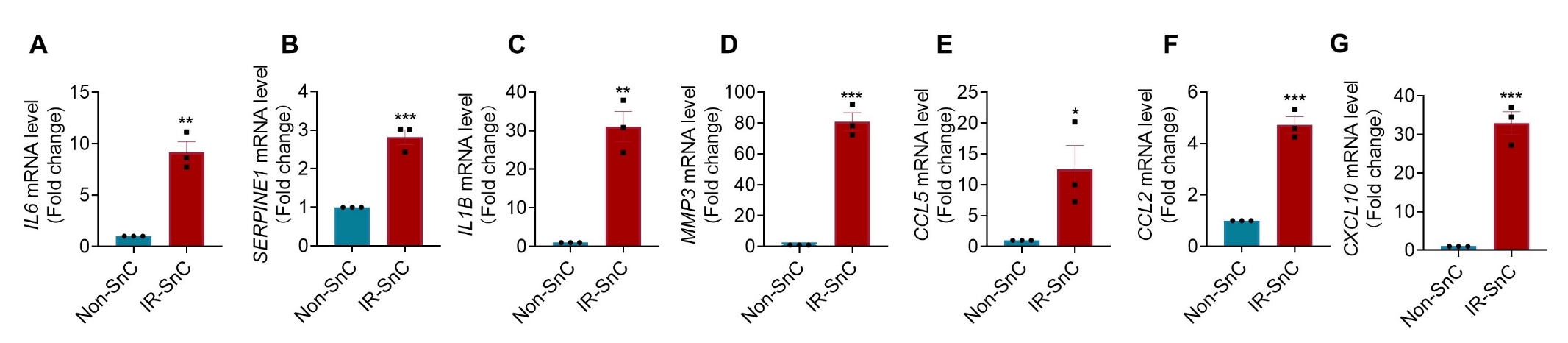
**

**Supplementary Fig. 1 mRNA levels of SASP factors in HEL Non-SnCs and IR-SnCs.** HEL fibroblasts at 70% confluence were exposed to 15 Gy. Cells became fully senescent 10 days after irradiation. mRNA levels of *IL6* (A), *SERPINE1* (B), *IL1β* (C), *MMP3* (D), *CCL5* (E), *CCL2* (F), and *CXCL10* (G) in HEL Non-SnCs and IR-SnCs were measured by qRT-PCR. Data are mean ± SEM (n = 3 independent experiments). Data were analyzed by two-sided Student’s *t*-test. **P* < 0.05, ***P* < 0.01, ****P* < 0.001, and *****P* < 0.0001 compared to Non-SnCs.


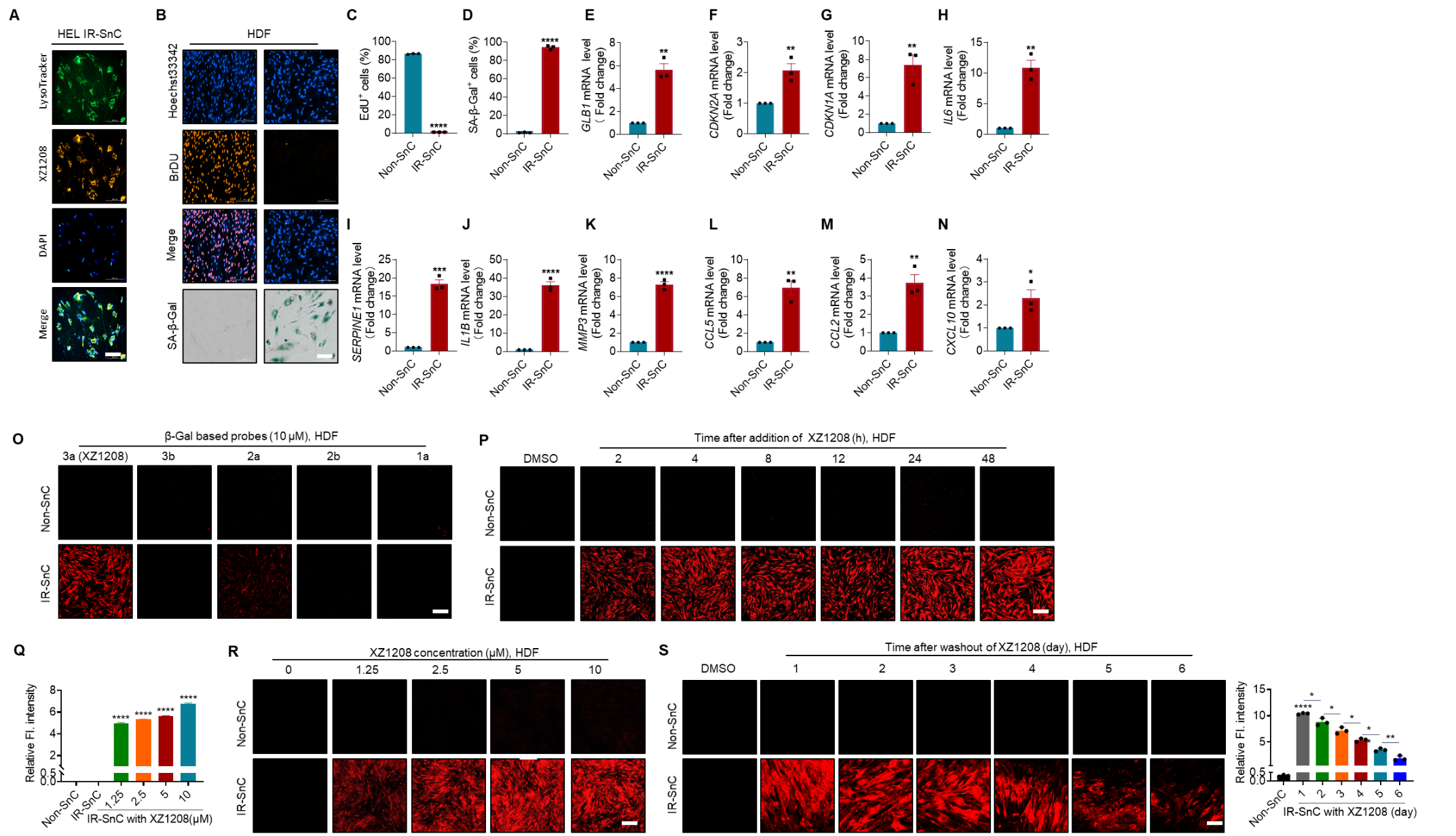


**Supplementary Fig. 2 Senescence markers and activation of fluorescence probes in IR-SnCs.** HEL and HDF cells at 70% confluence were exposed to 15 Gy. Cells became fully senescent 10 days after irradiation. (A) Intracellular localization of XZ1208 and LysoTracker in HEL IR-SnCs. (B) DNA synthesis and β-Gal staining in HDF Non-SnCs and IR-SnCs. (C) Quantification of EdU-positive HDF Non-SnCs and IR-SnCs. (D) Quantification of SA-β-Gal-positive HDF Non-SnCs and IR-SnCs. (E-N) mRNA levels of *GLB1*, *CDKN2A*, *CDKN1A*, *IL6*, *SERPINE1*, *IL1β*, *MMP3*, *CCL5*, *CCL2*, and *CXCL10* in HDF Non-SnCs and IR-SnCs. (O) Confocal imaging of HDF Non-SCs and IR-SnCs at 48 h after addition of indicated probes (10 µM). (P) Confocal imaging of HDF Non-SCs and IR-SnCs at different time points after addition of XZ1208 (10 µM). (Q) Quantification of fluorescence intensity in HEL Non-SnCs and IR-SnCs in Fig. 2I. (R) Confocal imaging of HDF Non-SCs and IR-SnCs at 48 h after addition of indicated concentrations of XZ1208. (S) XZ1208 (10 µM) was added to HDF Non-SCs and IR-SnCs for 48 h, with cells washed and imaged by laser scanning confocal microscopy at indicated time points. Representative images and quantification are presented. Data are mean ± SEM (n = 3 independent experiments). Representative images and quantification are presented. Scale bars, 200 μm (for A, B, and S) and 100 μm (for O, P, and R). Data were analyzed by two-sided Student’s *t*-test. **P* < 0.05, ***P* < 0.01, ****P* < 0.001, and *****P* < 0.0001 compared to Non-SnCs or indicated groups.

**
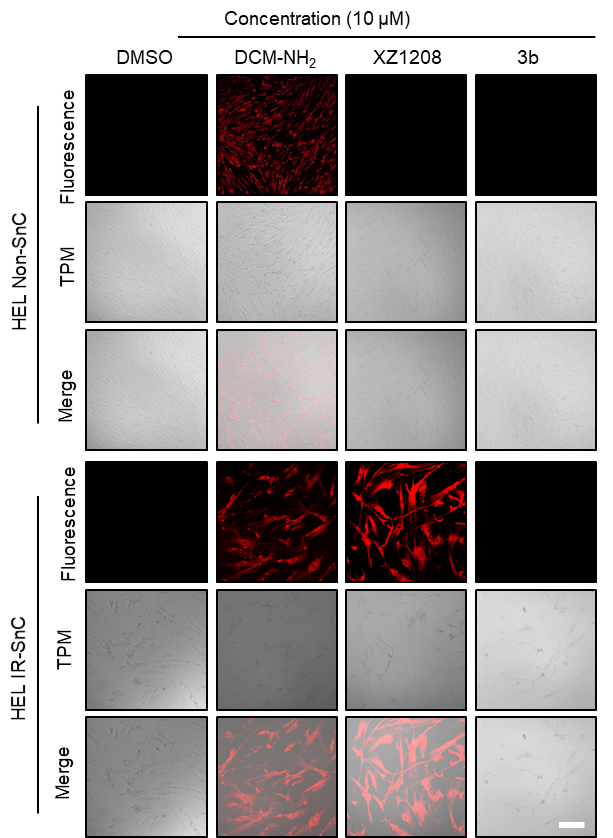
**

**Supplementary Fig. 3 Confocal images of HEL Non-SnCs and IR-SnCs after incubation with** **β-Gal probe XZ1208, free fluorophore** **DCM-NH_2_, or negative control probe 3b.** Cells were incubated with 10 µM XZ1208, DCM-NH_2,_ and **3b**, respectively, at 37 ℃ for 48 h. Scale bar, 200 μm.


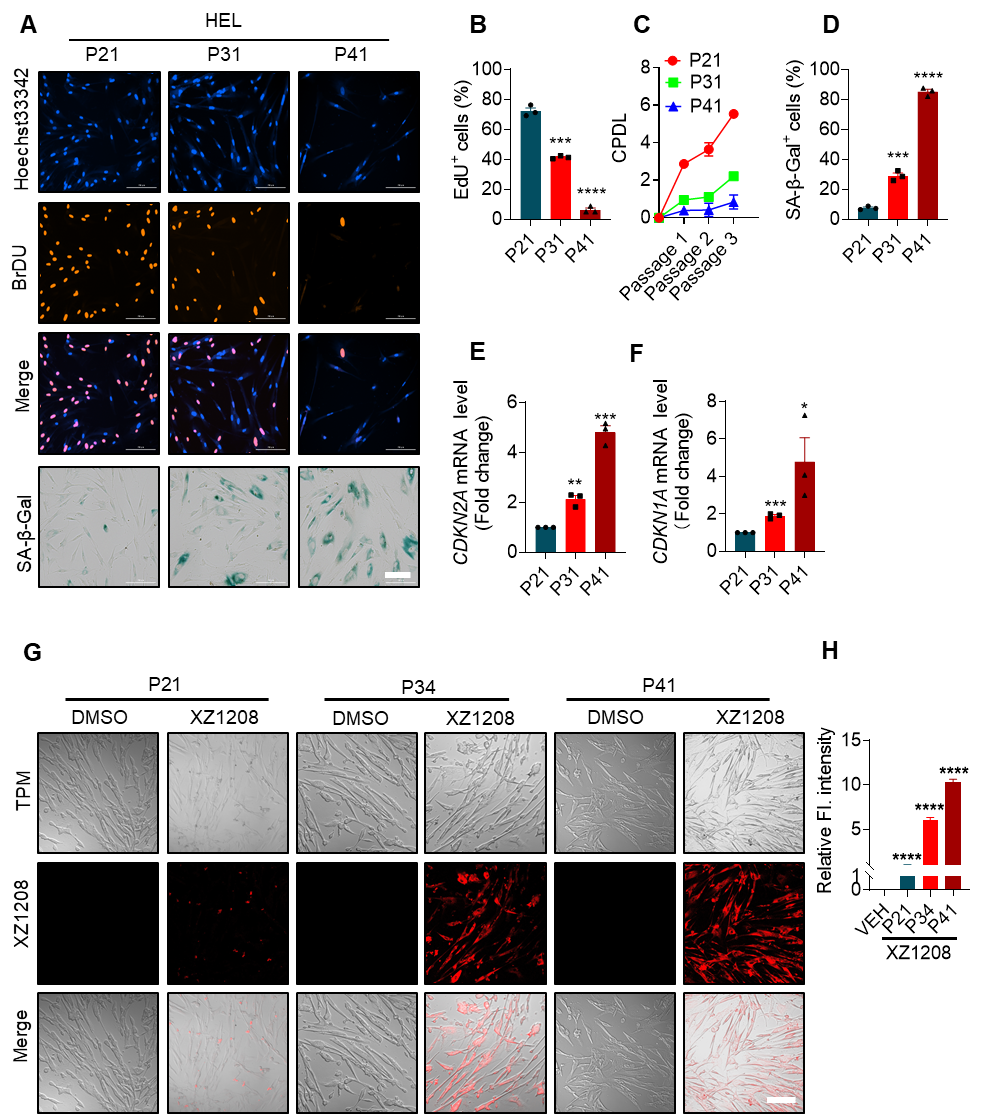


**Supplementary Fig. 4 Activation of XZ1208 in replicative senescent HEL fibroblasts.** (A) DNA synthesis and β-Gal staining in HEL Non-SnCs and REP-SnCs. (B) Quantification of EdU-positive HEL Non-SnCs and REP-SnCs. (C) CPDL of HEL Non-SnCs and REP-SnCs. (D) Quantification of SA-β-Gal-positive HEL Non-SnCs and REP-SnCs. (E-F) mRNA levels of *CDKN2A* and *CDKN1A* in HEL Non-SCs and REP-SnCs. (G-H) Confocal imaging and fluorescence intensity quantification of HEL Non-SCs and REP-SnCs at 48 h after addition of XZ1208 (10 µM). Data are mean ± SEM (n = 3 independent experiments). Scale bars, 200 μm. Representative images and quantification are presented. Data were analyzed by one-way ANOVA followed by Dunnett’s multiple comparisons test. **P* < 0.05, ***P* < 0.01, ****P* < 0.001, and *****P* < 0.0001 compared to P21 or VEH.

**
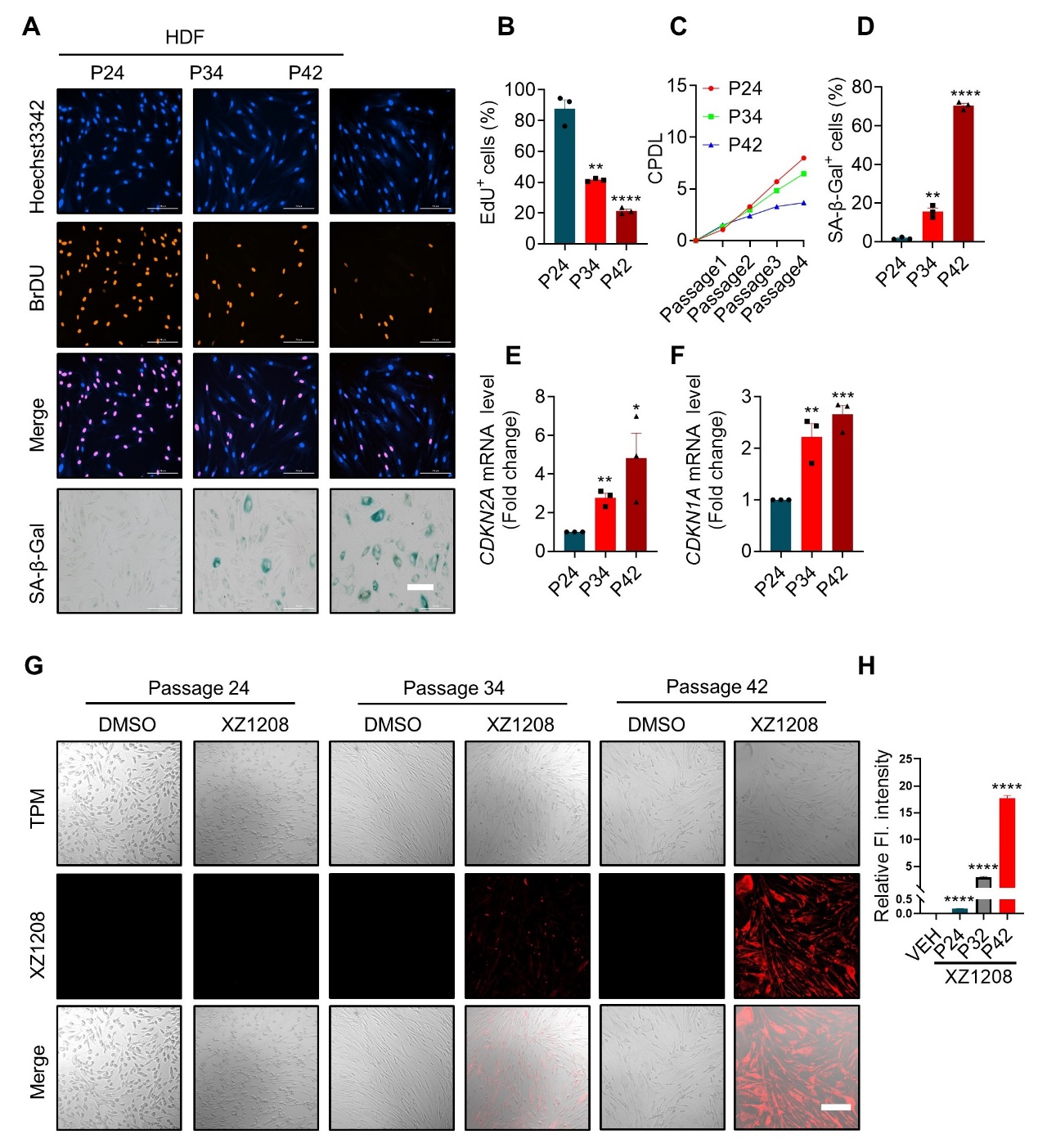
**

**Supplementary Fig. 5 Activation of XZ1208 in replicative senescent HDF cells.** (A) DNA synthesis and β-Gal staining in HDF Non-SnCs and REP-SnCs. (B) Quantification of EdU-positive cells in HDF Non-SnCs and REP-SnCs. (C) CPDL in HDF Non-SnCs and REP-SnCs. (D) Quantification of SA-β-Gal-positive cells in HDF Non-SnCs and REP-SnCs. (E-F) mRNA levels of *CDKN2A* and *CDKN1A* in HDF Non-SnCs and REP-SnCs. (G-H) Confocal imaging and fluorescence intensity quantification of HDF Non-SnCs and REP-SnCs at 48 h after addition of XZ1208 (10 µM). Data are mean ± SEM (n = 3 independent experiments). Representative images and quantification are presented. Scale bars, 200 μm. Data were analyzed by one-way ANOVA followed by Dunnett’s multiple comparisons test. **P* < 0.05, ***P* < 0.01, ****P* < 0.001, and *****P* < 0.0001 compared to P24 or VEH.


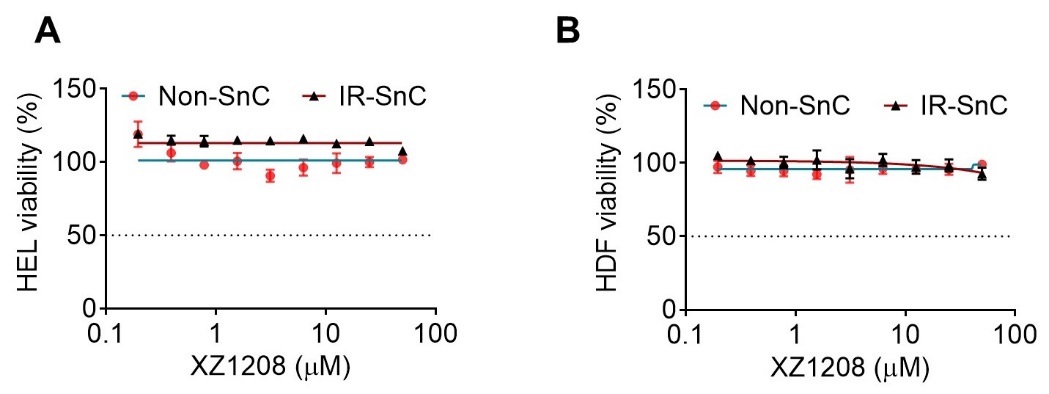


**Supplementary Fig. 6 Effects of XZ1208 on cell viability of Non-SnCs and IR-SnCs.** (A) HEL Non-SnCs and IR-SnCs were treated with indicated concentrations of XZ1208 for 72 h, and cell viability was measured by MTS assay. (B) HDF Non-SnCs and IR-SnCs were treated with indicated concentrations of XZ1208 for 72 h, and cell viability was measured by MTS assay.


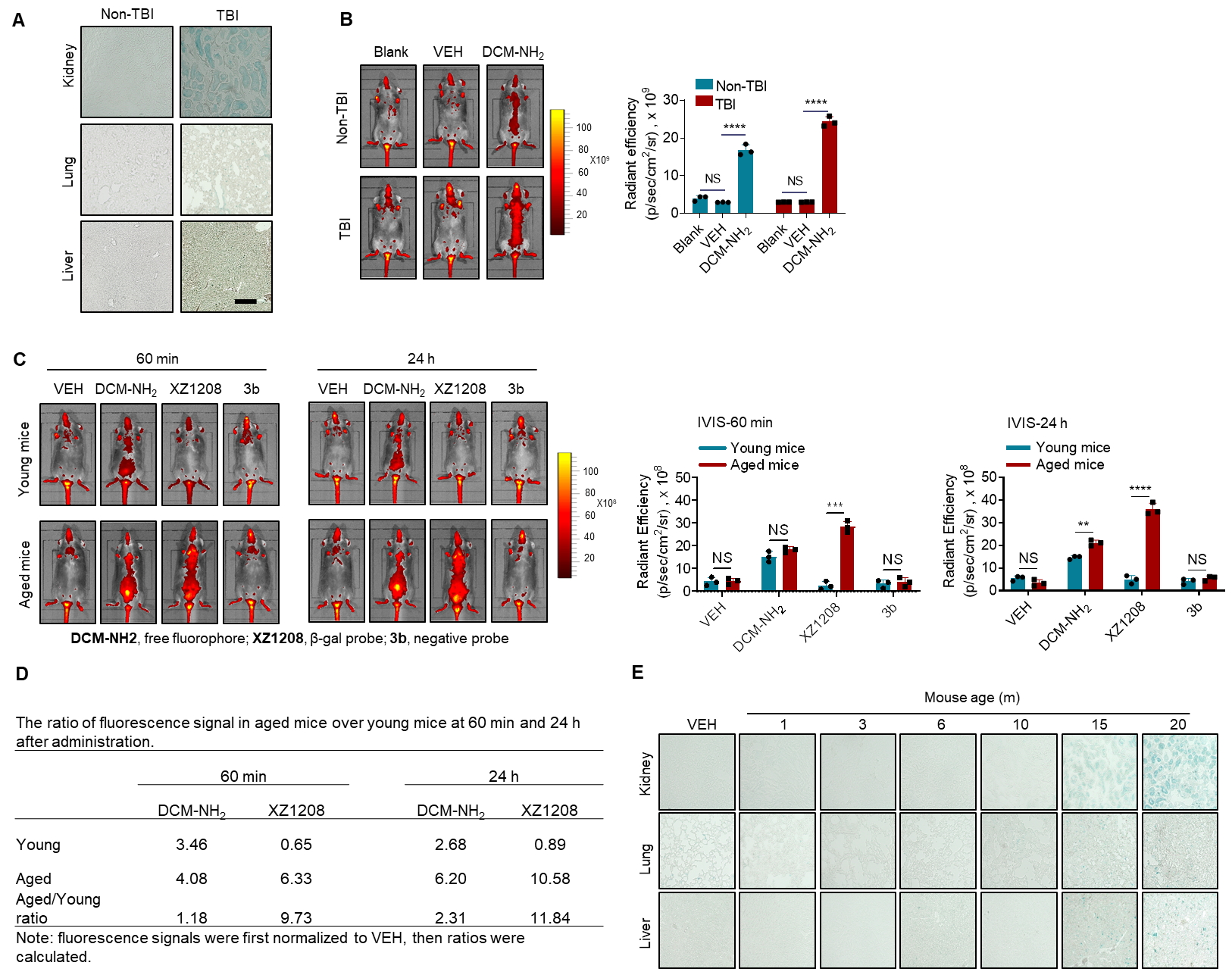


**Supplementary Fig. 7 β-Gal staining and imaging in mice**. (A) β-Gal staining of kidney, lung, and liver tissues of Non-TBI and TBI-mice. (B) Images and quantification of Non-TBI and TBI-mice. Mice were injected with vehicle (VEH) or 5 µM free fluorophore DCM-NH2 via the tail vein and imaged 24 h using the IVIS imaging system after administration (n = 3 mice for each group). (C) Images and quantification of young and naturally aged mice. Mice were injected with vehicle (VEH) or 5 µM free fluorophore DCM-NH2 via the tail vein and imaged at 60 min and 24 h using the IVIS imaging system after administration (n = 3 mice for each group). (D) The ratio of fluorescence signal in aged mice over young mice at 60 min and 24 h after administration of DCM-NH2 or XZ1208. (E) β-Gal staining of kidney, lung, and liver in different aged mice. *****P* < 0.0001 compared to VEH. NS, not significant. Scale bar, 200 μm.


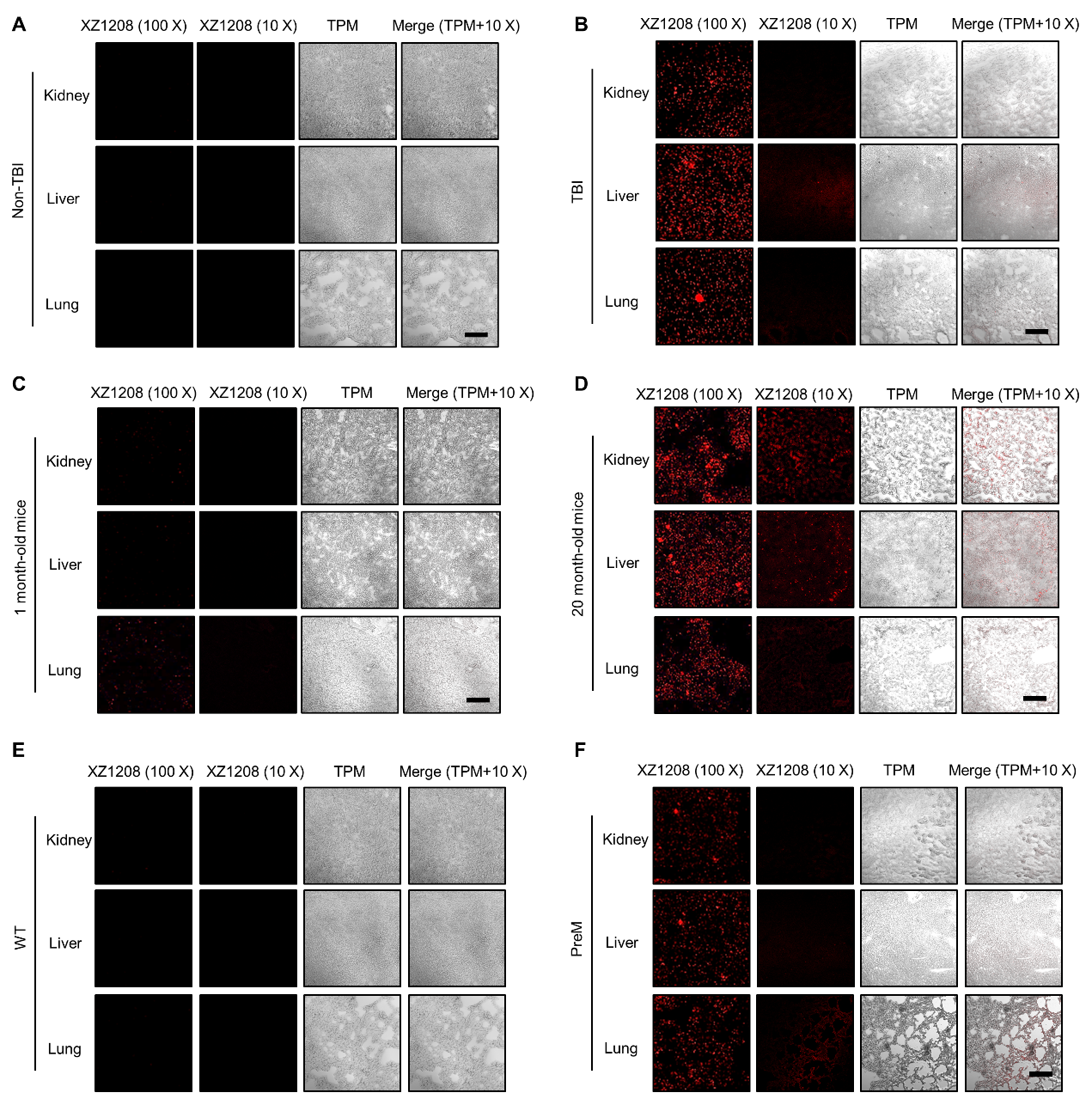


**Supplementary Fig. 8 Confocal images of major tissue sections of mice.** Mice were injected with 5 µM XZ1208 via the tail vein, and tissues were collected and imaged 24 h after administration. (A-B) Confocal images of Non-TBI and TBI mouse tissue sections. (C-D) Confocal images of young and naturally aged mouse tissue sections. (E-F) Confocal images of wild-type (WT) and prematurely aged (PreM) mice. To clearly show fluorescence signals, images at 10 × were magnified to 100 ×. Scale bars, 200 μm.


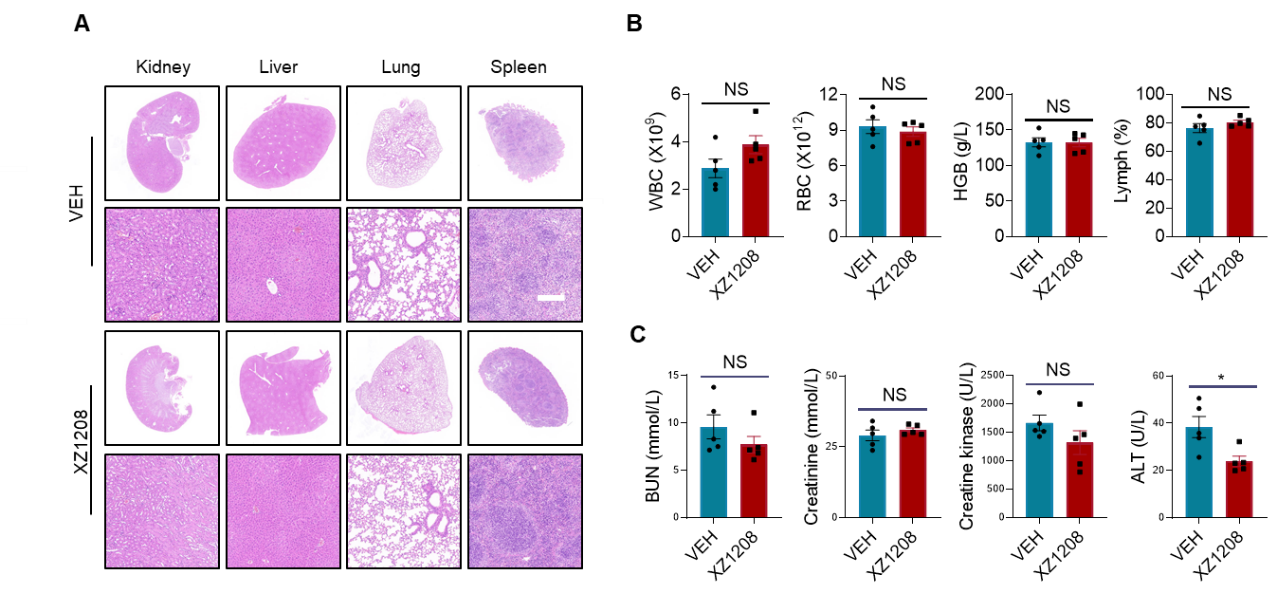


**Supplementary Fig. 9 H&E staining of major tissues and blood parameters in mice**. Young mice aged 2–3 months were intravenously injected with 10 µM XZ1208 via the tail vein. Blood and tissue samples were collected 7 days after administration. (A) H&E staining of kidney, liver, lung, and spleen of mice was performed. (B-C) White blood cells (WBC), red blood cells (RBC), lymphocytes (Lymph), hemoglobin (HGB) levels, blood urea nitrogen (BUN), creatinine, creatine kinase, and alanine aminotransferase (ALT) were measured after XZ1208 injection (n = 5 mice per group). Data were analyzed by two-sided Student’s *t*-test. **P* < 0.05 compared to vehicle (VEH) group. NS, not significant. Scale bars, 200 μm.


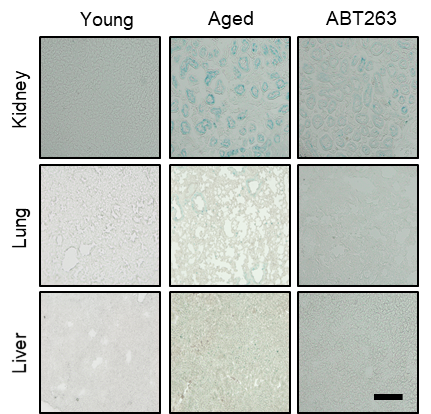


**Supplementary Fig. 10 β-Gal staining of major tissues in mice treated with ABT263.** Naturally aged mice were treated with ABT263, as described in the Materials and Methods section. Kidney, lung, and liver tissues were collected and stained for β-Gal. Scale bars, 200 μm.


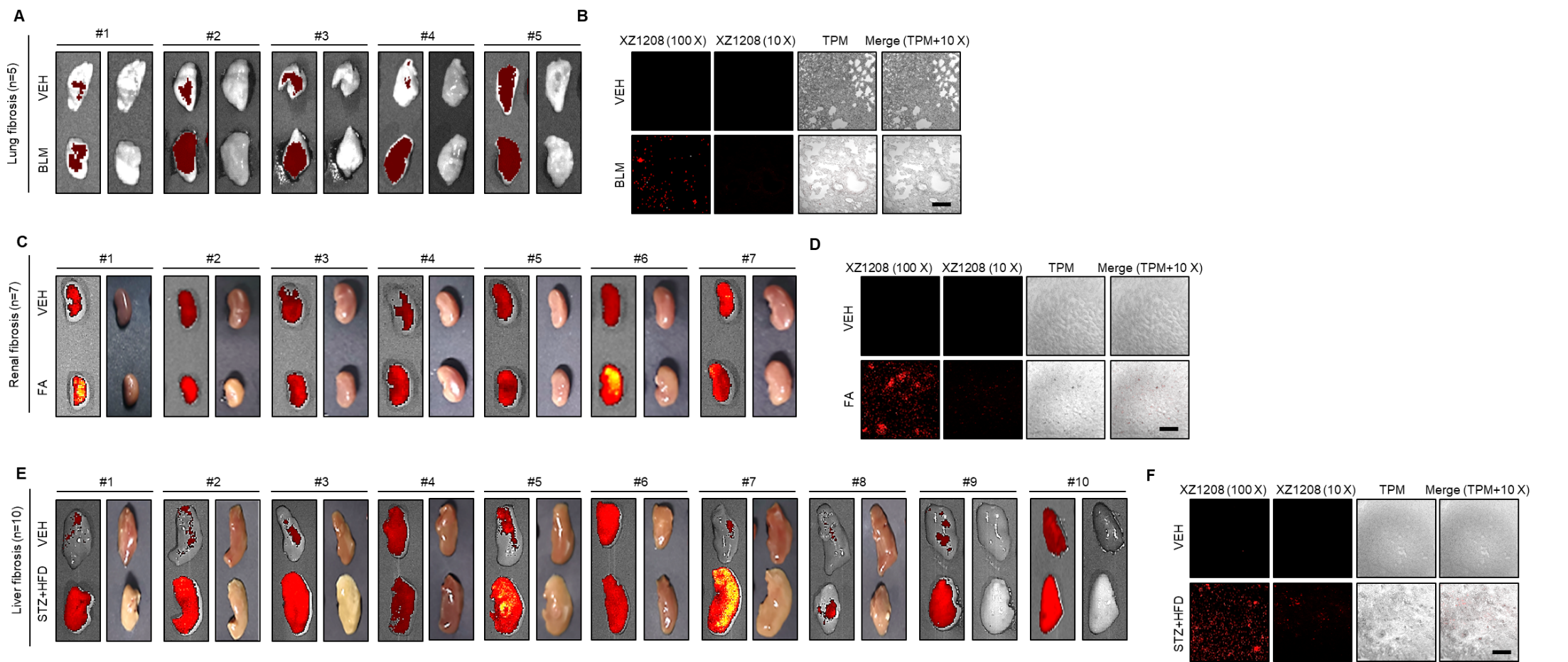


**Supplementary Fig. 11 Images of lung, kidney, and liver in fibrosis mouse models. (**A-B**)** Lung fibrosis was induced by administering mice with 3 mg/kg BLM via nasal inhalation. Lungs and lung tissue sections were imaged (n = 5 mice for each group). **(**C-D**)** Kidney fibrosis was induced by administering mice with a single dose of 250 mg/kg folic acid intraperitoneally. Kidneys and kidney tissue sections were imaged (n = 7 mice for each group). (E-F) Liver fibrosis was induced by injecting neonatal mice with 200 μg of STZ subcutaneously, followed by a high-fat diet (HFD). Liver tissues and sections were imaged (n = 10 mice for each group I). Tissues were imaged using the IVIS imaging system, and tissue sections were imaged using confocal microscopy. To clearly show fluorescence signals, images at 10 × were magnified to 100 ×. Scale bars, 200 μm.

**
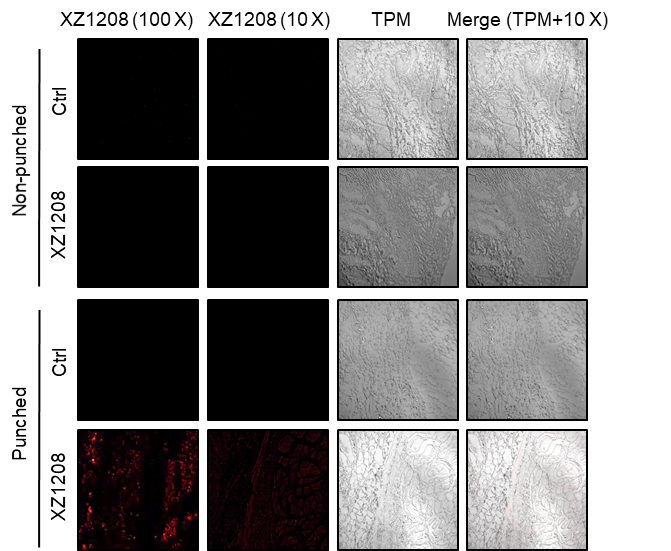
**

**Supplementary Fig. 12 Confocal images of skin in wound healing models.** Dorsal skin of mice was wounded using 4-mm punches, and skin tissues were collected and imaged using the IVIS imaging system on day 9 after intravenous injection of 5 µM XZ1208 via the tail vein. To clearly show fluorescence signals, images at 10 × were magnified to 100 ×. Scale bars, 200 μm.

| **Supplementary Table 1. Primers used for qRT-PCR analysis.** | |  |
| --- | --- | --- |
| **Name** | **Forward primer** | **Reverse primer** |
| *ACTB* | AGAGCTACGAGCTGCCTGAC | AGCACTGTGTTGGCGTACAG |
| *CDKN2A* | GAGCAGCATGGAGCCTTC | CCGCTGCAGACCCTCTAC |
| *CDKN1A* | TGTCCGTCAGAACCCATGC | AAAGTCGAAGTTCCATCGCTC |
| *IL1B* | AGCTACGAATCTCCGACCAC | CGTTATCCCATGTGTCGAAGAA |
| *CCL2* | CAGCCAGATGCAATCAATGCC | TGGAATCCTGAACCCACTTCT |
| *IL6* | ACTCACCTCTTCAGAACGAATTG | CCATCTTTGGAAGGTTCAGGTTG |
| *SERPINE1* | TGGTTCTGCCCAAGTTCTCC | CACCGTGCCACTCTCGTTC |
| *MMP3* | CACTCACAGACCTGACTCGG | GAGTCAGGGGGAGGTCCATAG |
| *CCL5* | TCATTGCTACTGCCCTCTGC | TCCTTGACCTGTGGACGACT |
| *GLB1* | ACGGTGGACTTTGGAACAG | TGATTGTGGAGTGAGGTTGG |
| *CXCL10* | CCACGTGTTGAGATCATTGCT | TGCATCGATTTTGCTCCCCT |
| *Actb* | CTAAGGCCAACCGTGAAAAG | ACCAGAGGCATACAGGGACA |
| *Cdkn2a* | TCCTCGCAGTTCGAATCTG | AACTCTTTCGGTCGTACCC |
| *Cdkn1a* | TCCACAGCGATATCCAGACA | GGACATCACCAGGATTGGAC |
| *Il1b* | TGTAATGAAAGACGGCACACC | TCTTCTTTGGGTATTGCTTGG |
| *Glb1* | TCCCACTGAACACTGAGGC | GGAGTATGAGGTCCGAAGAAT |
| *Mmp3* | CGATGGACAGAGGATGTCAC | CAGCCTTGGCTGAGTGGT |
| *Mmp13* | AAGGGGATAACAGCCACTACAA | ACCAACATAAAAATTAAGCCAAATG |
| *Tnf* | TTGTCTTAATAACGCTGATTTGGT | GGGAGCAGAGGTTCAGTGAT |
| *Il6* | GCTACCAAACTGGATATAATCAGGA | CCAGGTAGCTATGGTACTCCAGAA |
| *Note: Uppercase indicates human genes, lowercase indicates mouse genes.* | | |
