# Supplementary material for: A rationally designed fluorescence probe achieves highly specific and long‐term detection of senescence in vitro and in vivo
Source: Aging Cell. 2023 Jun 13;22(8):e13896. doi: 10.1111/acel.13896 (PMC10410003; doi:10.1111/acel.13896)
Supplement: Supplementary file 2 — Supporting Information File S2. [file ACEL-22-e13896-s001.docx]

**Supplementary Notes for Chemical Synthesis and Properties**

**A rationally designed fluorescence probe achieves highly specific and long-term detection of senescence *in vitro* and *in vivo***

Li Hu^1,2,3#^, Chanjuan Dong^4#^, Zhe Wang^5^, Shengyuan He^4^, Yiwen Yang^5^, Meiting Zi^1,2^, Huiqin Li^1,2^, Yanghuan Zhang^6^, Chuanjie Chen^3,4^, Runzi Zheng^6^, Shuting Jia^6^，Jing Liu^6^, Xuan Zhang^3,4,5*^, Yonghan He^1,2,3*^

^1^Key Laboratory of Healthy Aging Research of Yunnan Province, Kunming Institute of Zoology, Chinese Academy of Sciences, Kunming, China

^2^State Key Laboratory of Genetic Resources and Evolution, Kunming Institute of Zoology, Chinese Academy of Sciences, Kunming, China

^3^University of Chinese Academy of Sciences, Beijing, China

^4^Drug Discovery & Development Center, Shanghai Institute of Materia Medica, Chinese Academy of Sciences, Shanghai, China

^5^School of Chinese Materia Medica, Nanjing University of Chinese Medicine, Nanjing, China

^6^Laboratory of Molecular Genetics of Aging and Tumor, Medical School, Kunming University of Science and Technology, Kunming, China

* **Corresponding authors**:

Yonghan He

E-mail: heyonghan@mail.kiz.ac.cn; Tel./Fax: +86-871-65118976

Xuan Zhang

E-mail: zhangxuan@simm.ac.cn, Tel: +86-21-68077845

Table of Contents

| 1. **Supplementary Note 1** Time-dependent cleavage of NIR fluorescent probes by β-Gal from bovine liver and *E. coli*. | S3 |
| --- | --- |
| 1. **Supplementary Note 2** Reactivity of XZ1208 under different pH conditions | S3 |
| 1. **Supplementary Note 3** Long-term stability study of XZ1208 | S3 |
| 1. **Scheme S1** Synthetic route for NIR fluorescent probes | S4 |
| 1. Experimental Section: Chemistry | S5 |
| 1. Measurement of fluorescence quantum efficiency | S12 |
| 1. **Supplementary Note 4-21** ^1^H, ^13^C NMR, and HRMS spectra of NIR fluorescent probes | S13 |
| 1. Reference | S19 |

**Fig. Note 1** Time-dependent cleavage of NIR fluorescent probes by β-Gal from bovine liver (A) and *E. coli*. (B).

**Fig. Note 2** Reactivity of XZ1208 under different pH conditions.

**Fig. Note 3** Long-term stability study of XZ1208. Quantification of stability profile of XZ1208 using percentage area under the curve from HPLC traces in PBS.

**Scheme S1.** Synthetic route for NIR fluorescent probes. *Reagents and conditions:*(a) aldehyde **S2**/**S4**, piperidine, ACN, reflux; (b) compound **S6**, THF, 0 ℃; (c) compound **S7**, Na_2_CO_3_, DMF, rt; (d) LiOH monohydrate, CH_3_OH, THF, and H_2_O, rt; (e) PBr_3_, DCM, 0 ℃; (f) compound **S3**, K_2_CO_3_, DMF, rt; (g) triphosgene, NaHCO_3_ (aq), DCM, 0 ℃; (h) compound **S8b**, DMAP, TEA, DCM, rt.

**Experimental Section: Chemistry**

**General Methods.** THF, DCM, toluene, and DMF were obtained via a solvent purification system by filtering through two columns packed with activated alumina and 4 Å molecular sieves, respectively. All other chemicals obtained from commercial sources were used without further purification. Flash chromatography was performed using silica gel (230–400 mesh) as the stationary phase. Reaction progress was monitored by LC-MS or thin-layer chromatography (silica-coated glass plates) and visualized by UV light. ^1^H NMR spectra were recorded in CDCl_3_ or DMSO-*d6* at 400 MHz or 600 MHz. Chemical shifts δ are given in ppm using tetramethylsilane as an internal standard. Multiplicities of NMR signals are designated as singlet (s), broad singlet (br s), doublet (d), doublet of doublets (dd), triplet (t), quartet (q), and multiplet (m). All final compounds for biological testing were of ≥95.0% purity as analyzed by LC-MS, performed on an Agilent LC/MSD system using a Waters ACQUITY UPLC BEH C18 column (1.7 µm, 50 x 2.1 mm) at 40 °C. Gradient elution was used for UHPLC with a mobile phase of acetonitrile and water containing 0.1% formic acid. High-resolution mass spectra (HRMS) were recorded on an Agilent G6520 Q-TOF mass spectrometer.

**(*E*)-2-(2-(4-Hydroxystyryl)-4*H*-chromen-4-ylidene)malononitrile (S3):** A mixture of compound **S1** (100 mg, 0.48 mmol), 4-hydroxybenzaldehyde **S2** (70 mg, 0.58 mmol), and piperidine (150 µL, 1.52 mmol) was refluxed in dry ACN (8 mL) under argon atmosphere for 12 h. Then the solvent was removed under reduced pressure, and the crude product was purified by column chromatography to afford the title compound as a brown solid (120 mg, yield 80%). ^1^H NMR (400 MHz, DMSO-*d*_6_) δ 10.13 (br s, 1H), 8.74 (dd, *J* = 8.4, 1.4 Hz, 1H), 7.95 – 7.90 (m, 1H), 7.82 – 7.77 (m, 1H), 7.70 (d, *J* = 15.9 Hz, 1H), 7.67 – 7.57 (m, 3H), 7.29 (d, *J* = 15.9 Hz, 1H), 6.97 (s, 1H), 6.88 – 6.83 (m, 2H). LC-MS (ESI): m/z 313.2 [M+H] ^+^.

**(*E*)-2-(2-(4-Aminostyryl)-4*H*-chromen-4-ylidene)malononitrile (S5):** A mixture of compound **S1** (1.0 g, 4.8 mmol), 4-aminobenzaldehyde **S4** (698 mg, 5.76 mmol), and piperidine (1.5 mL, 15.2 mmol) was refluxed in dry ACN (30 mL) under argon atmosphere for 2 h. Then the solvent was removed under reduced pressure, and the crude product was crystallized in Et_2_O to afford the title compound as a brown solid (1.26 g, yield 84%). ^1^H NMR (400 MHz, DMSO-*d*_6_) δ 8.73 (dd, *J* = 8.4, 1.4 Hz, 1H), 7.92 – 7.87 (m, 1H), 7.78 (dd, *J* = 8.5, 1.3 Hz, 1H), 7.68 – 7.61 (m, 1H), 7.62 – 7.57 (m, 1H), 7.50 (d, *J* = 8.5 Hz, 2H), 7.10 (d, *J* = 15.7 Hz, 1H), 6.88 (s, 1H), 6.62 (d, *J* = 8.5 Hz, 2H). LC-MS (ESI): m/z 312.2 [M+H] ^+^.

**4-Nitrophenyl (*E*)-(4-(2-(4-(dicyanomethylene)-4*H*-chromen-2-yl)vinyl)phenyl)carbamate (S7):** To a solution of compound **S5** (500 mg, 1.60 mmol) in THF (20 mL) at 0 ℃ was added compound **S6** (510 mg, 2.54 mmol). The mixture was stirred at 0 ℃ overnight. Then the solvent was removed under reduced pressure, and the crude product was purified by column chromatography to afford the title compound as a red solid (215 mg, yield 28%). ^1^H NMR (600 MHz, DMSO-*d*_6_) δ 10.77 (s, 1H), 8.74 (dd, *J* = 8.3, 1.5 Hz, 1H), 8.35 – 8.31 (m, 2H), 7.96 – 7.91 (m, 1H), 7.83 – 7.76 (m, 3H), 7.74 (d, *J* = 16.0 Hz, 1H), 7.65 – 7.61 (m, 3H), 7.59 – 7.56 (m, 2H), 7.43 (d, *J* = 16.0 Hz, 1H), 7.02 (s, 1H). LC-MS (ESI): m/z 477.2 [M+H] ^+^.

**(2*R*,3*S*,4*S*,5*R*,6*S*)-2-(Acetoxymethyl)-6-(4-((((4-((*E*)-2-(4-(dicyanomethylene)-4*H*-chromen-2-yl)vinyl)phenyl)carbamoyl)oxy)methyl)-2-nitrophenoxy)tetrahydro-2*H*-pyran-3,4,5-triyl triacetate (2b):** A mixture of compound **S7** (100 mg, 0.21 mmol), compound **S8a** (160 mg, 0.32 mmol), and Na_2_CO_3_ (56 mg, 0.53 mmol) in dry DMF (6 mL) was stirred at room temperature under N_2_ overnight. The reaction mixture was poured into water and extracted with ethyl acetate. The organic phase was collected and washed with water x1, brine x1, dried over Na_2_SO_4_, filtered, and evaporated to dryness. The residue was purified by column chromatography to afford the title compound as a red solid (58 mg, yield 33%). ^1^H NMR (400 MHz, CDCl_3_) δ 8.91 (d, J = 7.1 Hz, 1H), 7.87 (d, J = 2.2 Hz, 1H), 7.74 (t, J = 7.8 Hz, 1H), 7.61 – 7.53 (m, 5H), 7.50 – 7.42 (m, 3H), 7.37 (d, J = 8.6 Hz, 1H), 6.92 (s, 1H), 6.85 (s, 1H), 6.74 (d, J = 15.9 Hz, 1H), 5.55 (dd, J = 10.5, 7.9 Hz, 1H), 5.47 (d, J = 3.3 Hz, 1H), 5.22 (s, 2H), 5.14 – 5.06 (m, 2H), 4.26 (dd, J = 11.3, 6.9 Hz, 1H), 4.16 (dd, J = 11.3, 6.2 Hz, 1H), 4.07 (t, J = 6.5 Hz, 1H), 2.19 (s, 3H), 2.13 (s, 3H), 2.07 (s, 3H), 2.02 (s, 3H). ^13^C NMR (125 MHz, CDCl_3_) δ 170.45, 170.29, 170.26, 169.52, 157.70, 152.98, 152.64, 152.50, 149.36, 141.50, 139.66, 138.24, 134.77, 133.61, 132.21, 130.30, 129.24, 126.12, 126.02, 125.11, 120.11, 118.99, 118.72, 118.03, 117.79, 116.95, 115.89, 106.84, 100.90, 71.64, 70.65, 67.98, 66.83, 65.52, 62.82, 61.44, 20.82, 20.78, 20.70. HRMS *m*/*z* calcd for C_42_H_36_N_4_NaO_15_ 859.2069, found 859.2075 [M+Na]^+^.

**3-Nitro-4-(((2*S*,3*R*,4*S*,5*R*,6*R*)-3,4,5-trihydroxy-6-(hydroxymethyl)tetrahydro-2*H*-pyran-2-yl)oxy)benzyl (4-((*E*)-2-(4-(dicyanomethylene)-4*H*-chromen-2-yl)vinyl)phenyl)carbamate (2a):** Compound **2b** (100 mg, 0.12 mmol) and LiOH monohydrate (30 mg, 0.71 mmol) was stirred in a mixture of CH_3_OH, THF, and H_2_O (2.5 mL, 3/2/1, v/v/v) for 2.5 h. Then the pH was adjusted to 4-5 with the addition of 5% citric acid (aq), and the mixture was concentrated under reduced pressure. The solid was collected and washed with water followed by ACN to give the title compound as an orange solid (67 mg, yield 84%). ^1^H NMR (600 MHz, DMSO-*d*_6_) δ 10.13 (s, 1H), 8.74 (d, J = 9.9 Hz, 1H), 7.96 (d, J = 2.1 Hz, 1H), 7.93 (m, 1H), 7.81 (d, J = 8.4 Hz, 1H), 7.75 – 7.69 (m, 4H), 7.62 (t, J = 7.6 Hz, 1H), 7.57 (d, J = 8.3 Hz, 2H), 7.46 (d, J = 8.8 Hz, 1H), 7.39 (d, J = 16.1 Hz, 1H), 7.01 (s, 1H), 5.18 (m, 3H), 5.06 (d, J = 7.7 Hz, 1H), 4.90 (d, J = 5.9 Hz, 1H), 4.66 (t, J = 5.6 Hz, 1H), 4.60 (d, J = 4.5 Hz, 1H), 3.70 (m, 1H), 3.64 (t, J = 6.3 Hz, 1H), 3.55 (m, 2H), 3.48 (m, 1H), 3.42 (m, 1H). ^13^C NMR (125 MHz, DMSO-*d*_6_) δ 158.47, 153.00, 152.90, 152.03, 149.34, 141.12, 139.89, 138.49, 135.36, 133.88, 130.06, 129.24, 129.19, 126.12, 124.62, 124.41, 119.04, 118.22, 117.64, 117.25, 117.11, 115.93, 106.24, 101.01, 75.81, 73.33, 69.98, 67.98, 64.58, 60.25, 59.70. HRMS *m*/*z* calcd for C_34_H_27_N_4_O_11_ 667.1682, found 667.1679 [M-H]^-^.

**(2*R*,3*S*,4*S*,5*R*,6*S*)-2-(Acetoxymethyl)-6-(4-(bromomethyl)-2-nitrophenoxy)tetrahydro-2*H*-pyran-3,4,5-triyl triacetate (S9):** To a solution of **S8a** (200 mg, 0.40 mmol) in CH_2_Cl_2_ (5 mL) at 0 ℃ was added PBr_3_ (57 µL, 0.60 mmol). The mixture was stirred at 0 ℃ for 1h and poured into water. The organic phase was collected and washed with water x1, brine x1, dried over Na_2_SO_4_, filtered, and evaporated to dryness. The residue was purified by column chromatography to afford the title compound as a white solid (149 mg, yield 66%). ^1^H NMR (600 MHz, CDCl_3_) δ 7.84 (d, J = 2.3 Hz, 1H), 7.55 (dd, J = 8.6, 2.3 Hz, 1H), 7.34 (d, J = 8.6 Hz, 1H), 5.55 (dd, J = 10.5, 7.9 Hz, 1H), 5.47 (dd, J = 3.5, 1.3 Hz, 1H), 5.12 – 5.09 (m, 2H), 4.47 (s, 2H), 4.26 (dd, J = 11.4, 7.0 Hz, 1H), 4.17 (dd, J = 11.4, 6.1 Hz, 1H), 4.08 (m,1H), 2.19 (s, 3H), 2.13 (s, 3H), 2.08 (s, 3H), 2.02 (s, 3H). LC-MS (ESI): m/z 562.1 [M+H] ^+^.

**(2*R*,3*S*,4*S*,5*R*,6*S*)-2-(Acetoxymethyl)-6-(4-((4-((*E*)-2-(4-(dicyanomethylene)-4*H*-chromen-2-yl)vinyl)phenoxy)methyl)-2-nitrophenoxy)tetrahydro-2*H*-pyran-3,4,5-triyl triacetate (1b):** A mixture of compound **S3** (200 mg, 0.64 mmol), compound **S9** (364 mg, 0.65 mmol), and K_2_CO_3_ (360 mg, 2.61 mmol) in dry DMF (6 mL) was stirred at room temperature under N_2_ overnight. The reaction mixture was poured into water and extracted with ethyl acetate. The organic phase was collected and washed with water x1, brine x1, dried over Na_2_SO_4_, filtered, and evaporated to dryness. The residue was purified by column chromatography to afford the title compound as a red solid (322 mg, yield 63%). ^1^H NMR (400 MHz, CDCl_3_) δ 8.93 (d, J = 8.3 Hz, 1H), 7.91 (d, J = 1.7 Hz, 1H), 7.74 (t, J = 7.8 Hz, 1H), 7.64 – 7.53 (m, 5H), 7.46 (t, J = 7.8 Hz, 1H), 7.42 – 7.38 (m, 1H), 7.02 (d, J = 8.5 Hz, 2H), 6.86 (s, 1H), 6.72 (d, J = 16.0 Hz, 1H), 5.56 (dd, J = 10.5, 7.9 Hz, 1H), 5.48 (d, J = 3.4 Hz, 1H), 5.15 – 5.06 (m, 4H), 4.28 (dd, J = 11.2, 6.8 Hz, 1H), 4.16 (dd, J = 11.4, 6.2 Hz, 1H), 4.08 (t, J = 6.6 Hz, 1H), 2.20 (s, 3H), 2.14 (s, 3H), 2.07 (s, 3H), 2.02 (s, 3H). ^13^C NMR (100 MHz, CDCl_3_) δ 170.42, 170.27, 170.23, 169.50, 160.13, 157.84, 152.93, 152.41, 149.13, 141.46, 138.41, 134.72, 132.65, 132.54, 129.87, 128.32, 126.03, 125.89, 124.16, 120.13, 118.67, 117.93, 116.99, 116.96, 115.94, 115.53, 106.50, 100.85, 71.56, 70.62, 68.41, 67.94, 66.80, 62.35, 61.40, 20.77, 20.74, 20.67. HRMS *m*/*z* calcd for C_41_H_35_N_3_NaO_14_ 816.2011, found 816.2018 [M+Na]^+^.

**2-(2-((*E*)-4-((3-Nitro-4-(((2*S*,3*R*,4*S*,5*R*,6*R*)-3,4,5-trihydroxy-6-(hydroxymethyl)tetrahydro-2*H*-pyran-2-yl)oxy)benzyl)oxy)styryl)-4*H*-chromen-4-ylidene)malononitrile** **(1a):** Compound **1b** (50 mg, 0.06 mmol) and LiOH monohydrate (18 mg, 0.43 mmol) was stirred in a mixture of CH_3_OH, THF, and H_2_O (2.5 mL, 3/2/1, v/v/v) for 3 h. Then the pH was adjusted to 4-5 with the addition of 5% citric acid (aq), and the mixture was concentrated under reduced pressure. The solid was collected and washed with water followed by ACN to give the title compound as an orange solid (33 mg, yield 84%). ^1^H NMR (400 MHz, DMSO-*d*_6_) δ 8.74 (dd, J = 8.4, 1.4 Hz, 1H), 7.99 (d, J = 2.2 Hz, 1H), 7.93 (m, 1H), 7.81 (dd, J = 8.6, 1.3 Hz, 1H), 7.79 – 7.71 (m, 4H), 7.62 (m, 1H), 7.47 (d, J = 8.8 Hz, 1H), 7.40 (d, J = 16.1 Hz, 1H), 7.15 (d, J = 8.8 Hz, 2H), 7.00 (s, 1H), 5.23 – 5.17 (m, 3H), 5.07 (d, J = 7.6 Hz, 1H), 4.93 (d, J = 5.9 Hz, 1H), 4.69 (t, J = 5.5 Hz, 1H), 4.63 (d, J = 4.5 Hz, 1H), 3.71 (t, J = 3.8 Hz, 1H), 3.65 (t, J = 6.1 Hz, 1H), 3.61 – 3.46 (m, 3H), 3.41 (m, 1H). ^13^C NMR (125 MHz, DMSO-*d*_6_) δ 159.95, 158.55, 152.90, 152.02, 149.31, 139.91, 138.53, 135.34, 133.46, 130.34, 130.07, 128.03, 126.10, 124.62, 124.03, 119.02, 117.33, 117.28, 117.22, 117.10, 115.94, 115.47, 106.12, 101.06, 75.80, 73.33, 69.99, 67.98, 67.89, 60.26, 59.59. HRMS *m*/*z* calcd for C_33_H_27_N_3_NaO_10_ 648.1589, found 648.1583 [M+Na]^+^.

**(*E*)-2-(2-(4-Isocyanatostyryl)-4*H*-chromen-4-ylidene)malononitrile (S10):** To a biphasic mixture of compound **S5** (300 mg, 0.96 mmol) in DCM (3 mL) and saturated NaHCO_3_ (aq) (3 mL) at 0 ℃ was added triphosgene (94 mg, 0.32 mmol) in one portion. The reaction mixture was stirred at 0 ℃ for 15 min and then extracted with DCM. The organic layer was collected and washed with water x1, brine x1, dried over Na_2_SO_4_, filtered, and evaporated to dryness to give the title compound as a red solid (320 mg, yield 99%), which was used directly in the next step.

**(2*R*,3*S*,4*S*,5*R*,6*S*)-2-(Acetoxymethyl)-6-(4-((((4-((*E*)-2-(4-(dicyanomethylene)-4*H*-chromen-2-yl)vinyl)phenyl)carbamoyl)oxy)methyl)phenoxy)tetrahydro-2*H*-pyran-3,4,5-triyl triacetate (3b):** A solution of **S10** (320 mg, 0.95 mmol) in DCM (3 mL) was added dropwise into a mixture of **S8b** (431 mg, 0.95 mmol), DMAP (58 mg, 0.47 mmol), and TEA (0.66 mL, 4.7 mmol) in DCM (3 mL). The reaction mixture was stirred at room temperature overnight. Then it was poured into saturated NH_4_Cl (aq) (10 mL) and extracted with DCM (3 x 10 mL). The organic phase was collected and washed with brine x1, dried over Na_2_SO_4_, filtered, and evaporated to dryness. The residue was purified by column chromatography to afford the title compound as a red solid (532 mg, yield 71%). ^1^H NMR (600 MHz, CDCl_3_) δ 8.92 (dd, J = 8.3, 1.4 Hz, 1H), 7.74 (m, 1H), 7.58 (d, J = 15.9 Hz, 1H), 7.55 (m, 3H), 7.49 – 7.44 (m, 3H), 7.37 (d, J = 8.6 Hz, 2H), 7.02 (d, J = 8.9 Hz, 2H), 6.86 (s, 1H), 6.79 (s, 1H), 6.74 (d, J = 15.9 Hz, 1H), 5.49 (dd, J = 10.4, 7.9 Hz, 1H), 5.46 (d, J = 3.5 Hz, 1H), 5.17 (s, 2H), 5.11 (dd, J = 10.4, 3.5 Hz, 1H), 5.05 (d, J = 7.9 Hz, 1H), 4.23 (dd, J = 11.3, 6.8 Hz, 1H), 4.16 (dd, J = 11.4, 6.5 Hz, 1H), 4.06 (t, J = 6.5 Hz, 1H), 2.19 (s, 3H), 2.07 (s, 6H), 2.02 (s, 3H). ^13^C NMR (100 MHz, CDCl_3_) δ 170.38, 170.24, 170.14, 169.39, 157.64, 157.06, 152.89, 152.85, 152.34, 139.94, 138.25, 134.62, 130.71, 130.18, 129.80, 129.08, 125.95, 125.84, 118.69, 118.57, 117.86, 117.36, 117.05, 116.86, 115.79, 106.60, 99.55, 71.08, 70.79, 68.60, 66.83, 65.42, 62.48, 61.32, 20.74, 20.68, 20.67, 20.59. HRMS *m*/*z* calcd for C_42_H_37_N_3_NaO_13_ 814.2219, found 814.2215 [M+Na]^+^.

**4-(((2*S*,3*R*,4*S*,5*R*,6*R*)-3,4,5-Trihydroxy-6-(hydroxymethyl)tetrahydro-2*H*-pyran-2-yl)oxy)benzyl (4-((*E*)-2-(4-(dicyanomethylene)-4*H*-chromen-2-yl)vinyl)phenyl)carbamate (3a, XZ1208):** Compound **3b** (20 mg, 0.025 mmol) and LiOH monohydrate (5.3 mg, 0.13 mmol) was stirred in a mixture of CH_3_OH, THF, and H_2_O (1.5 mL, 3/2/1, v/v/v) for 3 h. Then the pH was adjusted to 4-5 with the addition of 5% citric acid (aq), and the mixture was concentrated under reduced pressure. The solid was collected and washed with water followed by ACN to give the title compound as an orange solid (5.0 mg, yield 32%). ^1^H NMR (400 MHz, DMSO-*d*_6_) δ 10.08 (s, 1H), 8.75 (dd, *J* = 8.3, 1.4 Hz, 1H), 7.97 – 7.90 (m, 1H), 7.84 – 7.80 (m, 1H), 7.76 – 7.68 (m, 3H), 7.65 – 7.60 (m, 1H), 7.59 – 7.54 (m, 2H), 7.44 – 7.35 (m, 3H), 7.05 (d, *J* = 8.4 Hz, 2H), 7.01 (s, 1H), 5.19 (d, *J* = 5.1 Hz, 1H), 5.12 (s, 2H), 4.91 – 4.82 (m, 2H), 4.68 – 4.63 (m, 1H), 4.52 (d, *J* = 4.6 Hz, 1H), 3.72 – 3.68 (m, 1H), 3.60 – 3.52 (m, 3H), 3.50 – 3.39 (m, 2H). ^13^C NMR (125 MHz, DMSO-*d*_6_) δ 158.50, 157.40, 153.22, 152.91, 152.04, 141.31, 138.55, 135.36, 129.90, 129.44, 129.24, 129.05, 126.12, 124.62, 119.04, 118.15, 117.54, 117.27, 117.11, 116.22, 115.94, 106.21, 100.85, 75.50, 73.29, 70.24, 68.12, 65.81, 60.37, 59.66. HRMS *m*/*z* calcd for C_34_H_28_N_3_O_9_ 622.1831, found 622.1830 [M-H]^-^.

**Measurement of fluorescence quantum efficiency**

Absorption and emission spectra of XZ1208 (1 μM), DCM-NH_2_ (1 μM), and fluorescein (1 μM) were used to calculate Ф_fl_ was determined in PBS buffer (with 30% DMSO, v/v, pH = 7.4), λ_ex_ = 442 nm. The Ф_fl_ of XZ1208 and DCM-NH_2_ was determined by comparison with the fluorescence of fluorescein in 0.1 M NaOH (Ф_fl,st_ = 0.95). The Ф_fl_ was calculated according to the following equation (Parker & Rees, 1960):

$$\Phi_{fl,x}=\Phi_{fl,st}\frac{F_{x}A_{st}}{F_{st}A_{x}}$$

where subscripts x and st represent the sample to be tested and the standard sample, respectively. F represents the integrated fluorescence emission. A represents the absorbance of compound at its respective excitation wavelengths.


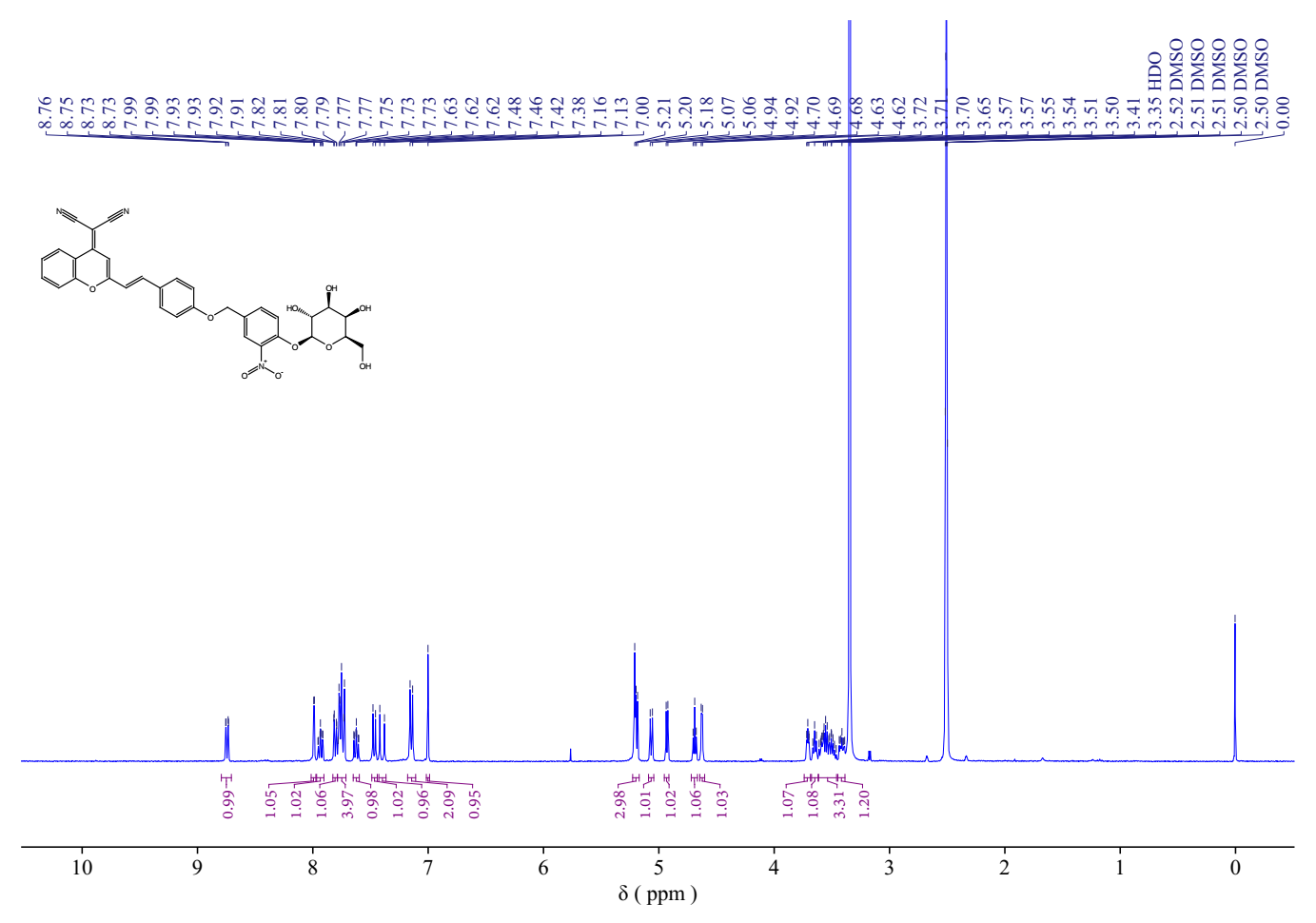


**Fig. Note 4** ^1^H-NMR spectrum of compound **1a**.


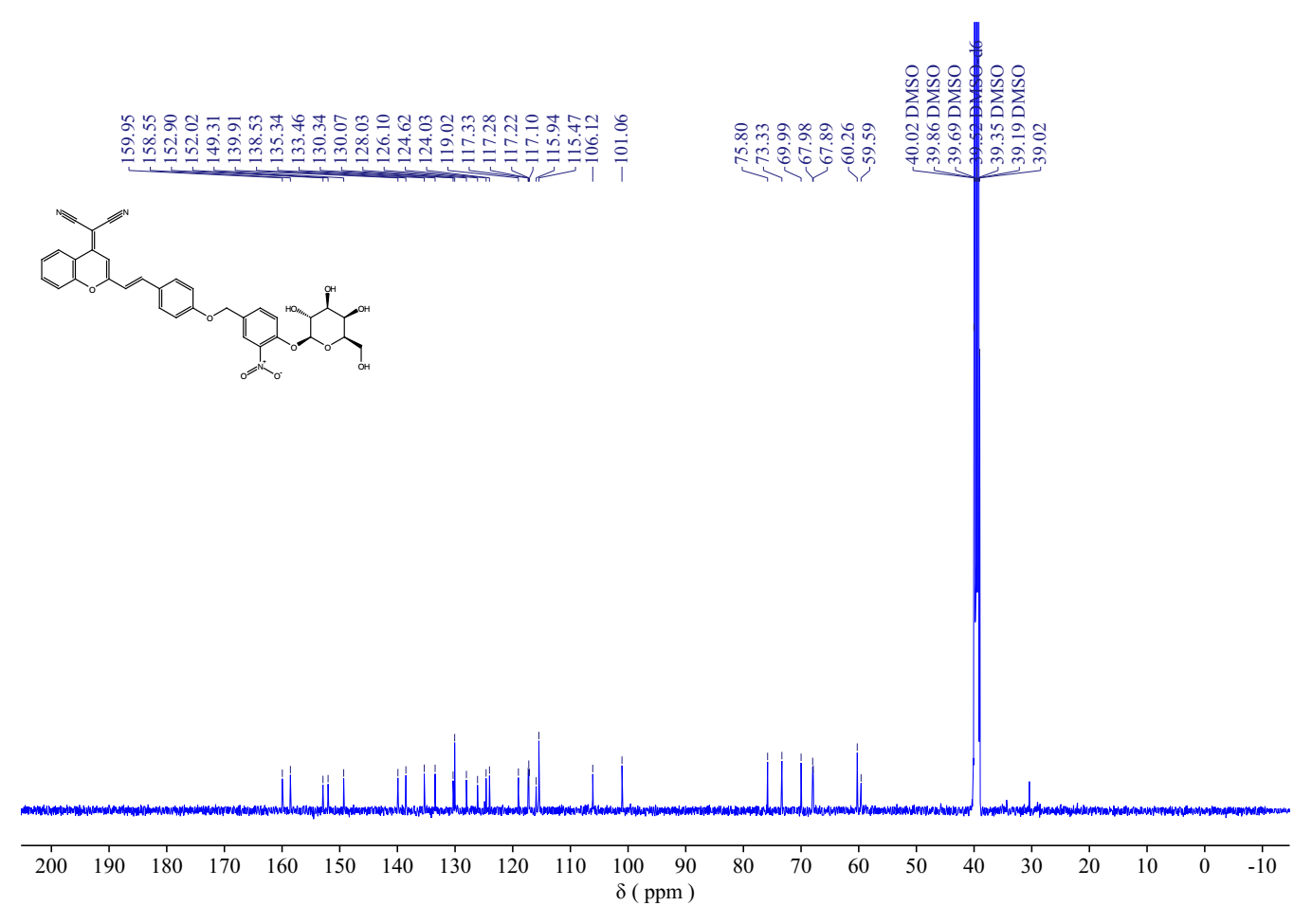


**Fig. Note 5** ^13^C-NMR spectrum of compound **1a**.


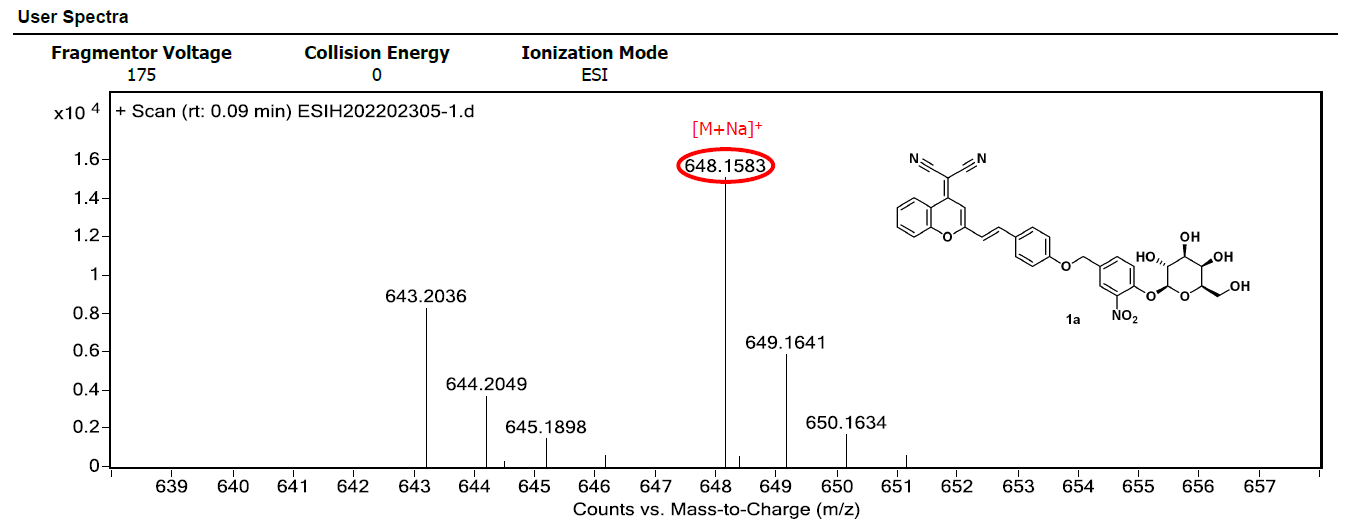


**Fig. Note 6** HRMS spectrum of compound **1a**.


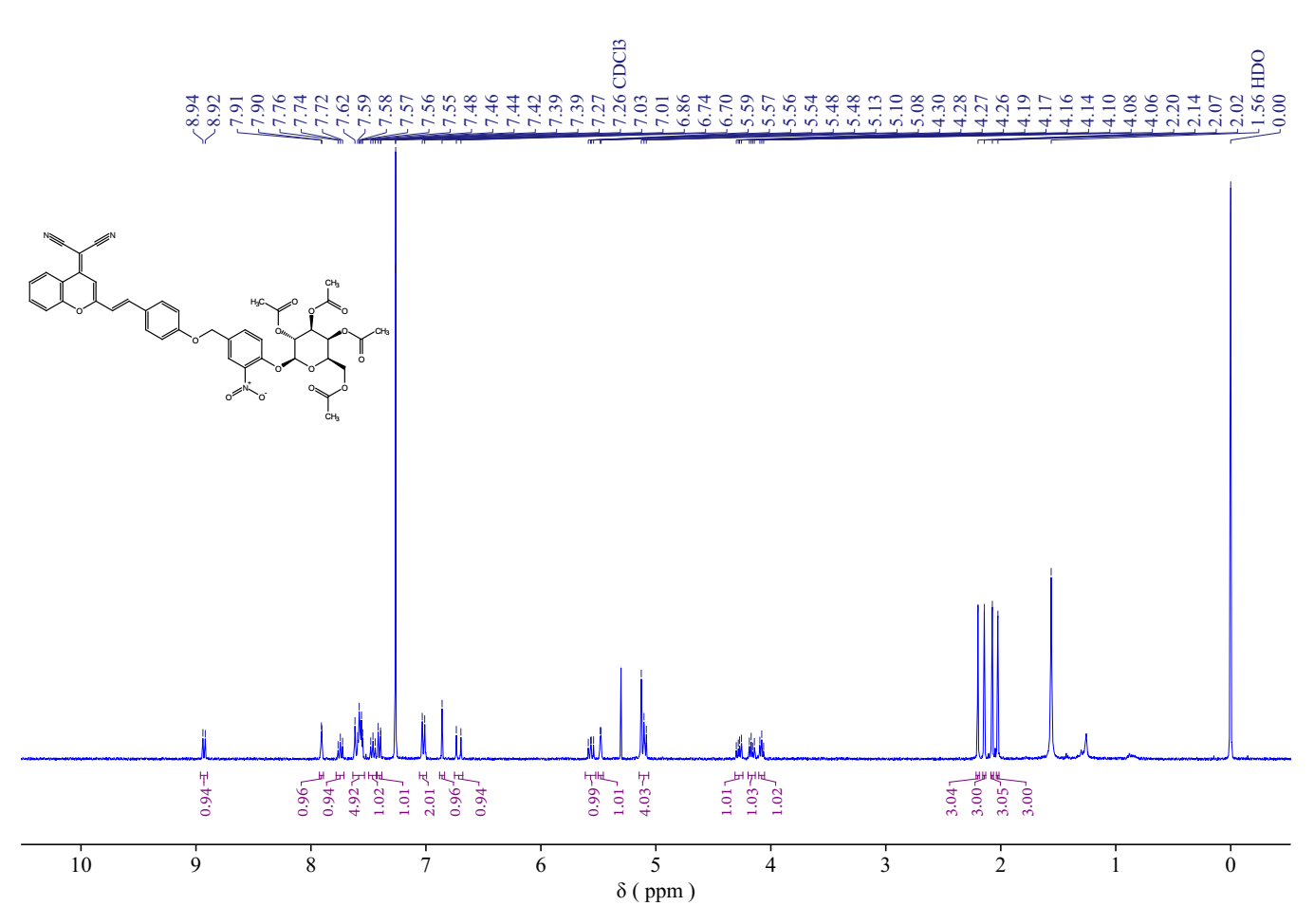


**Fig. Note 7** ^1^H-NMR spectrum of compound **1b**.


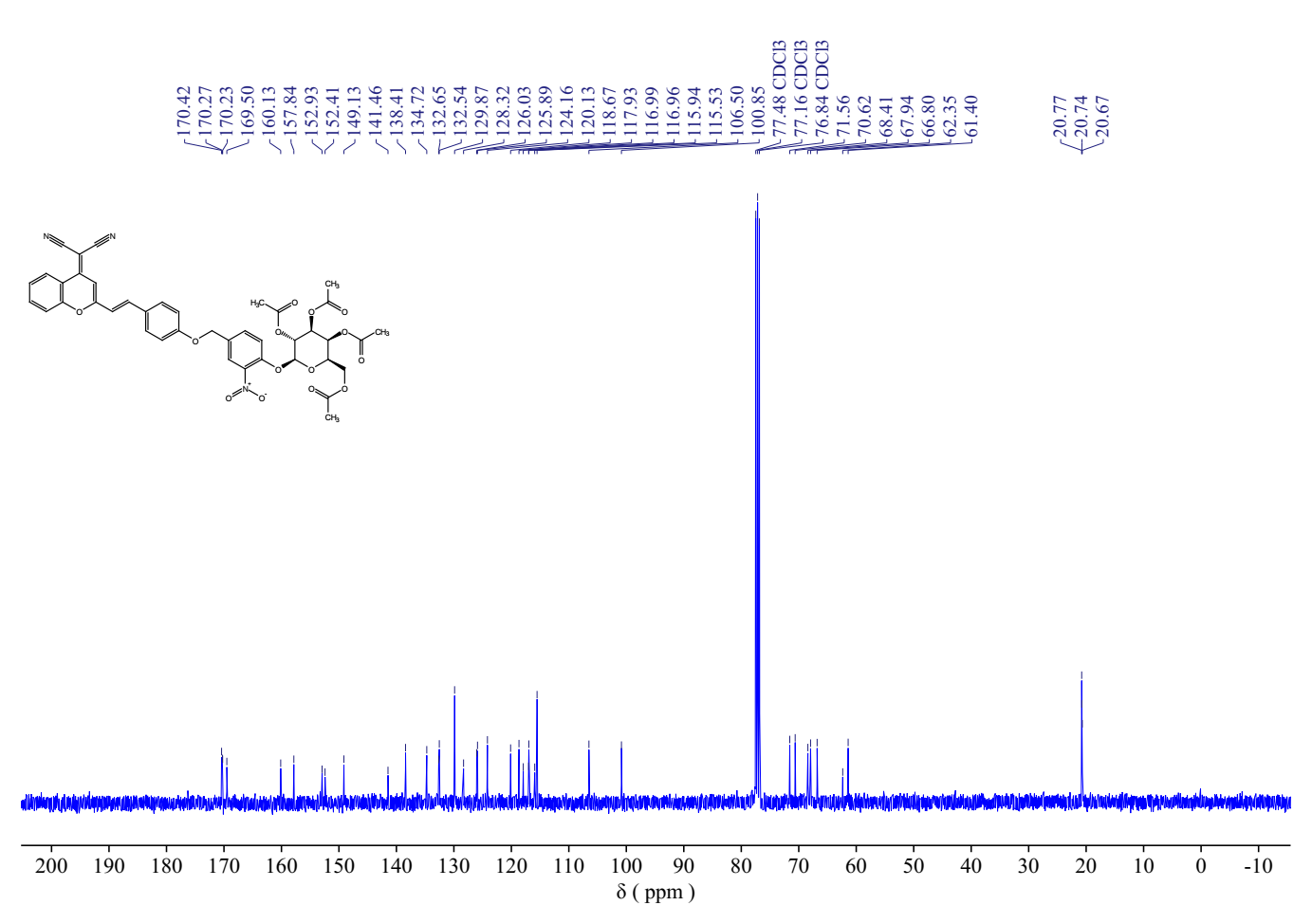


**Fig. Note 8** ^13^C-NMR spectrum of compound **1b**.


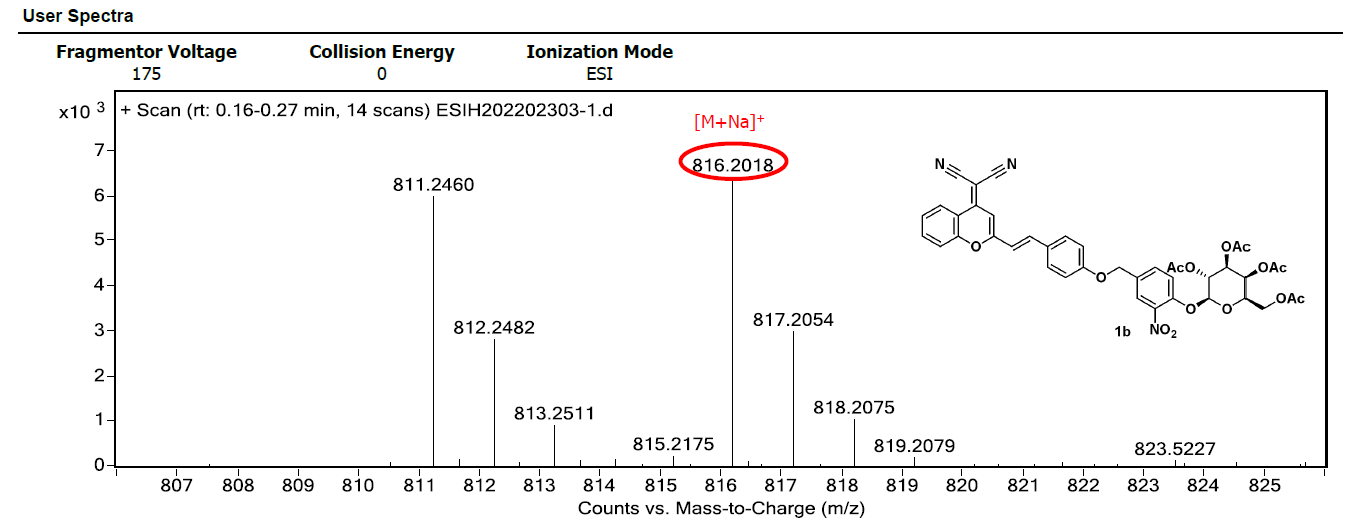


**Fig. Note 9** HRMS spectrum of compound **1b**.


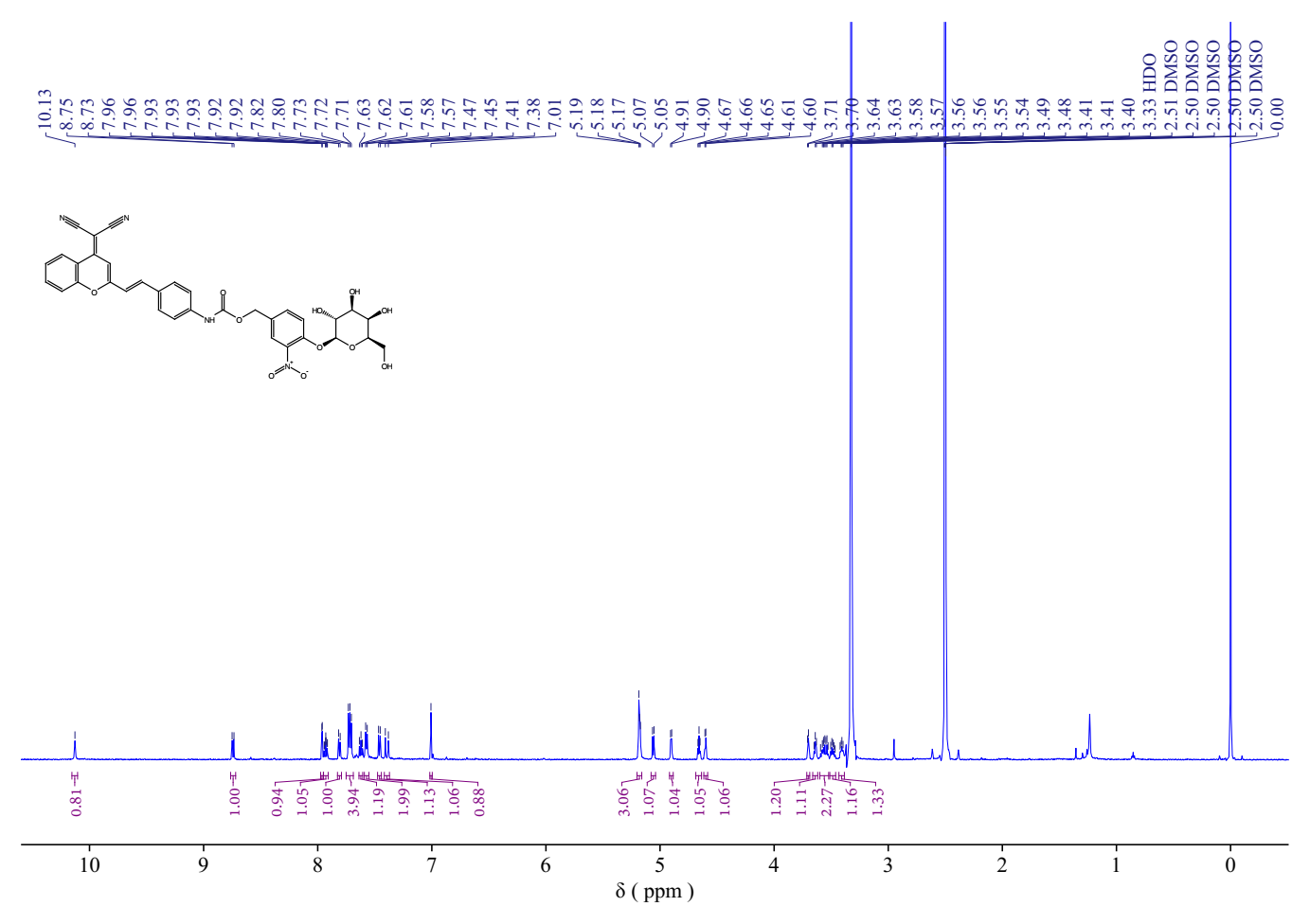


**Fig. Note 10** ^1^H-NMR spectrum of compound **2a**.


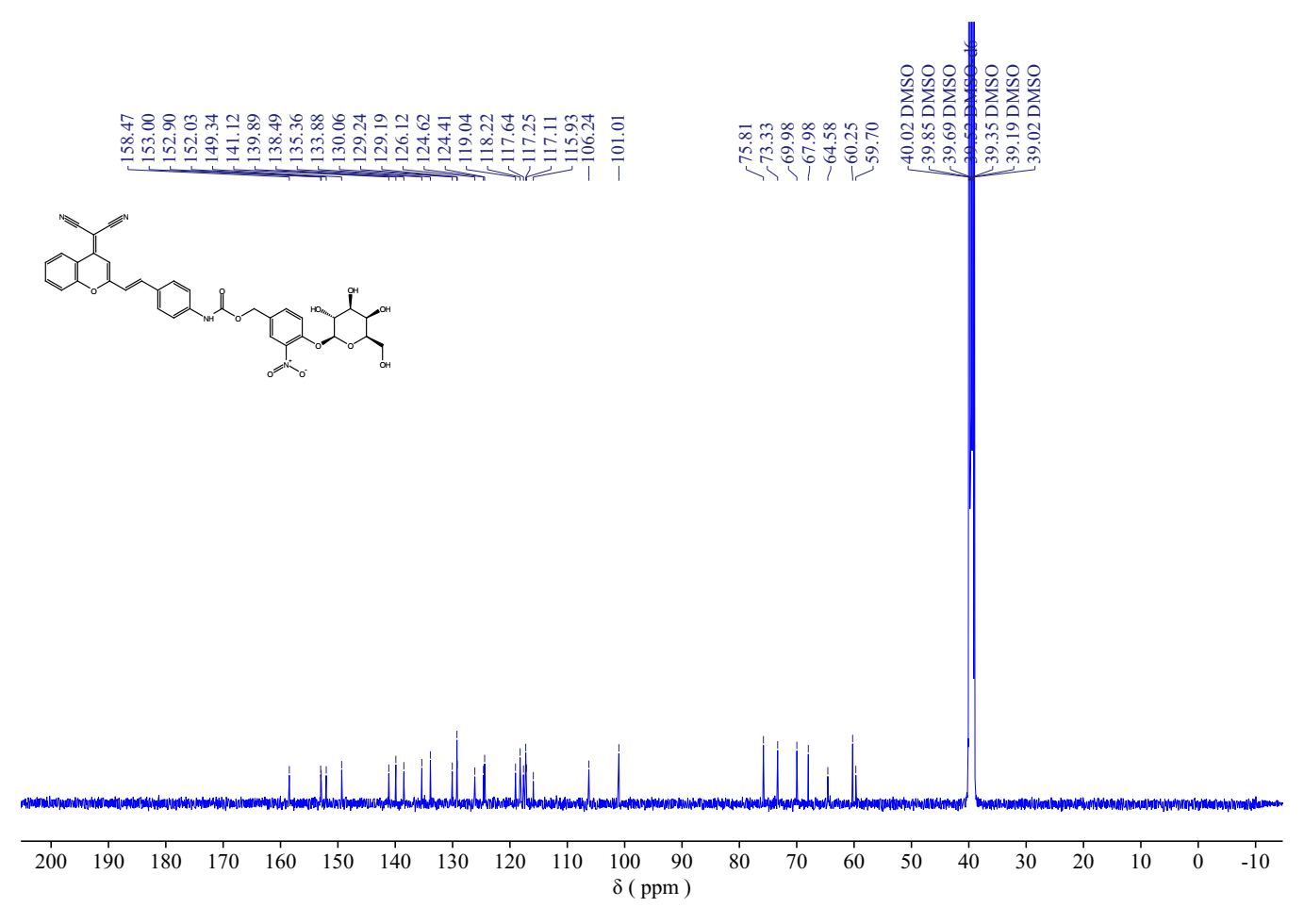


**Fig. Note 11** ^13^C-NMR spectrum of compound **2a**.


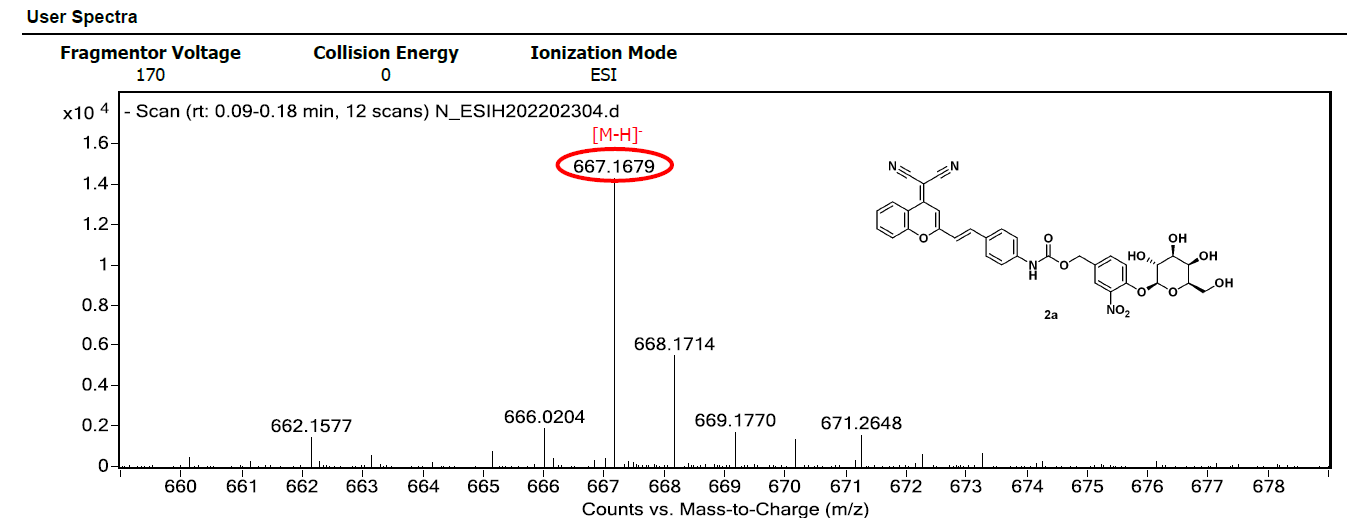


**Fig. Note 12** HRMS spectrum of compound **2a**.


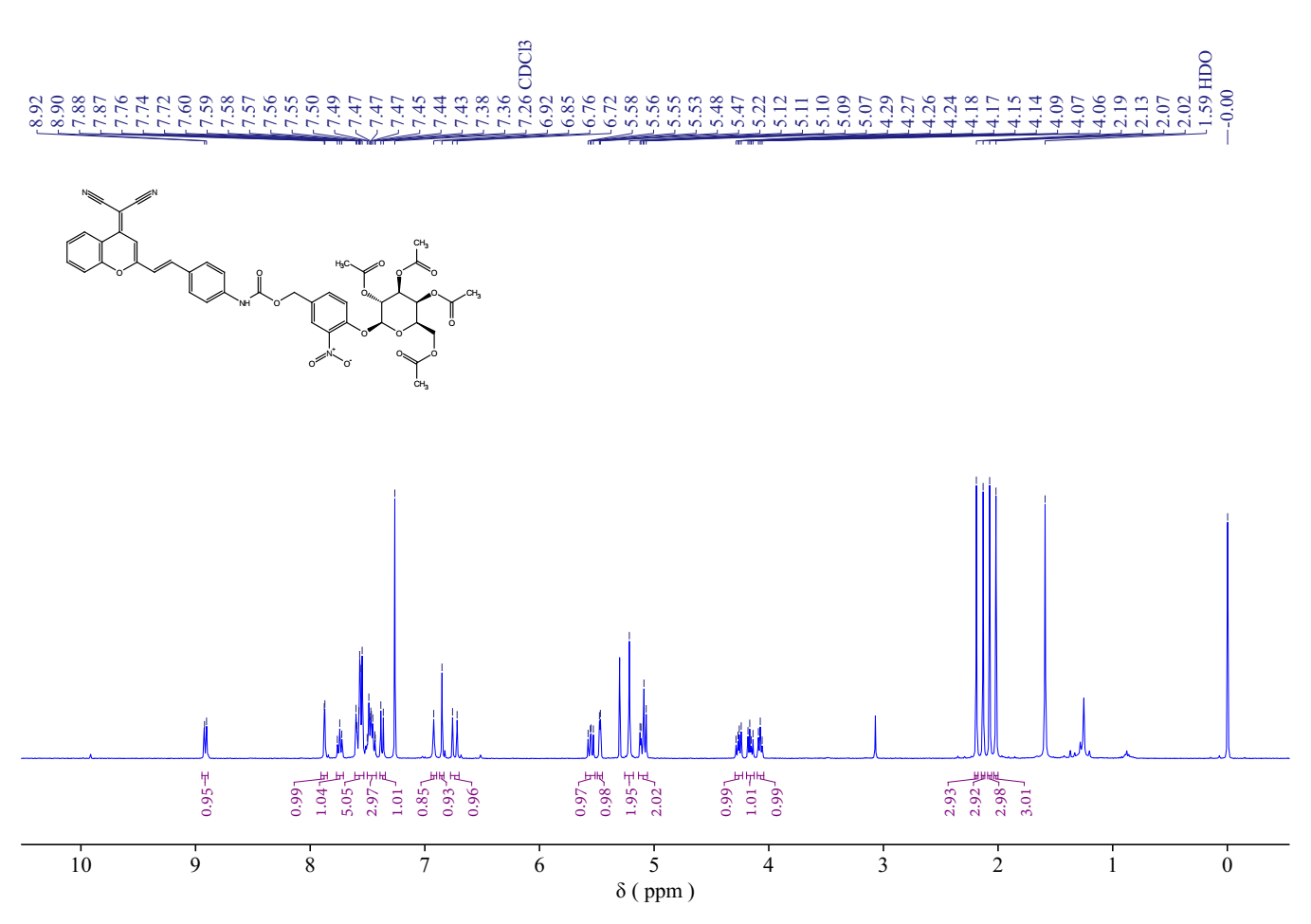


**Fig. Note 13** ^1^H-NMR spectrum of compound **2b**.


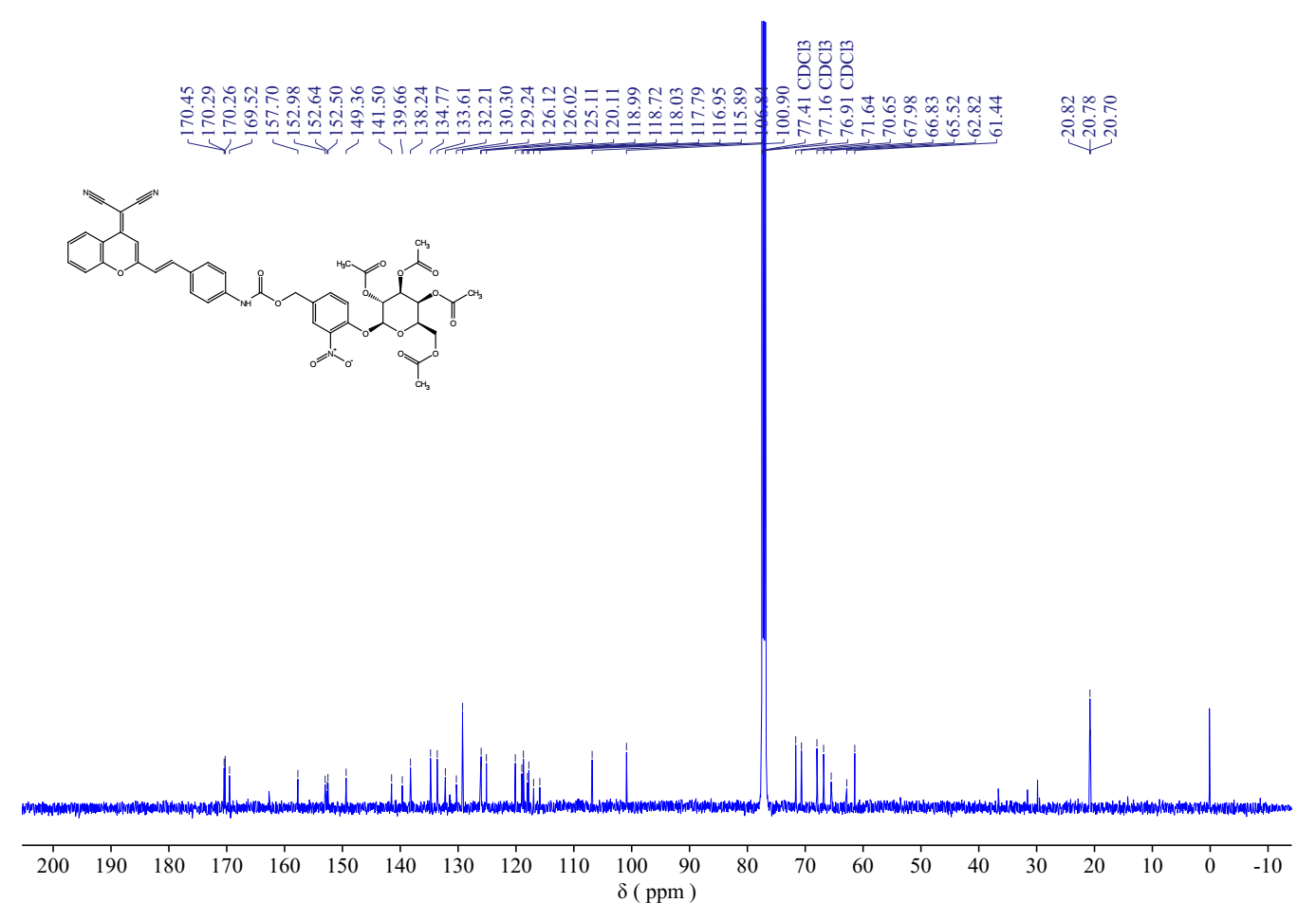


**Fig. Note 14** ^13^C-NMR spectrum of compound **2b**.


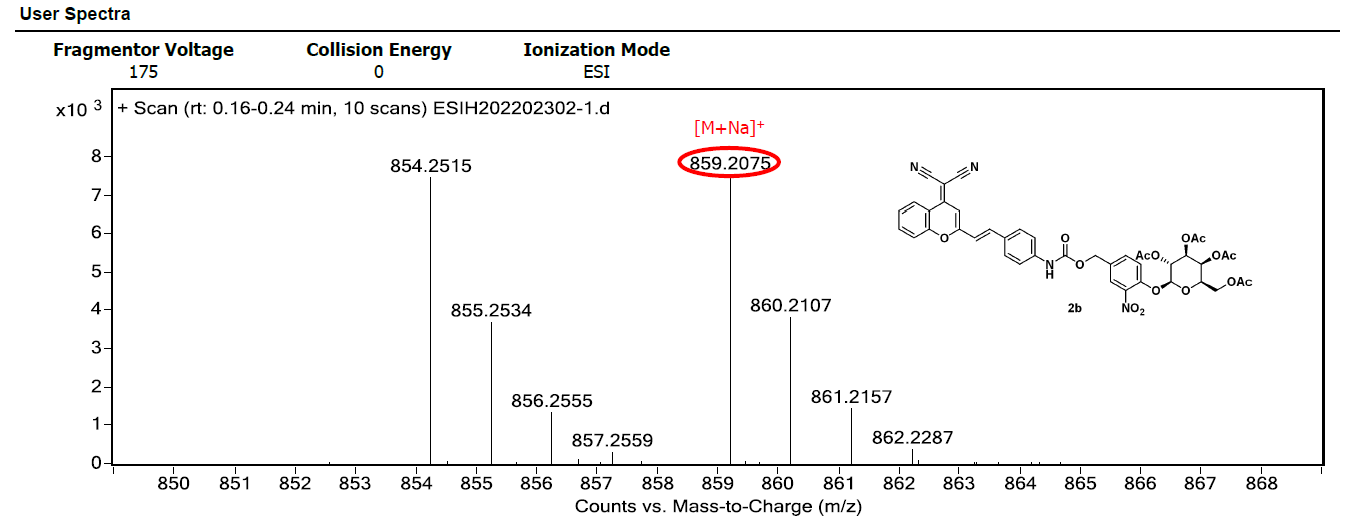


**Fig. Note 15** HRMS spectrum of compound **2b**.


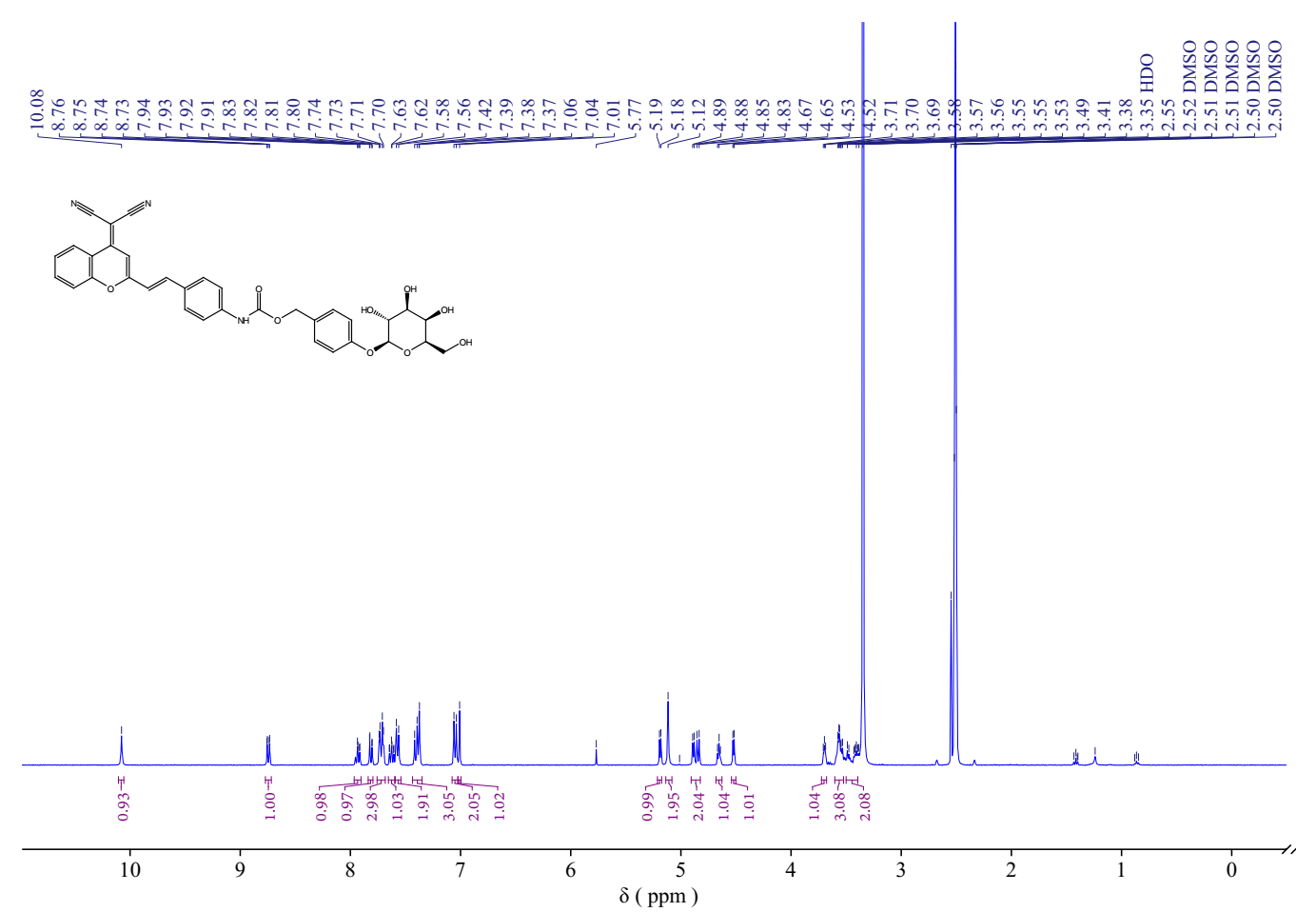


**Fig. Note 16** ^1^H-NMR spectrum of **XZ1208**.


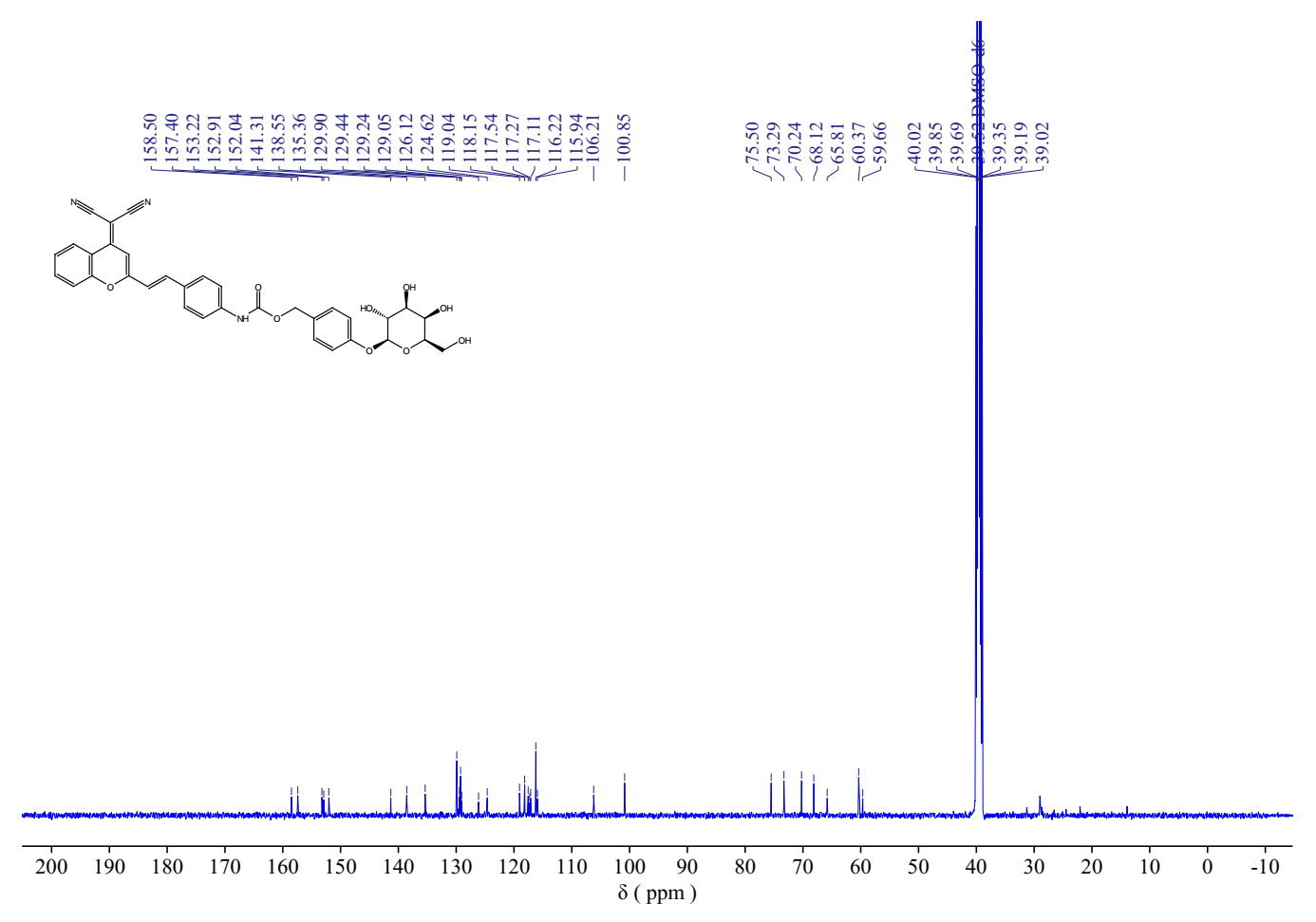


**Fig. Note 17** ^13^C-NMR spectrum of **XZ1208**.


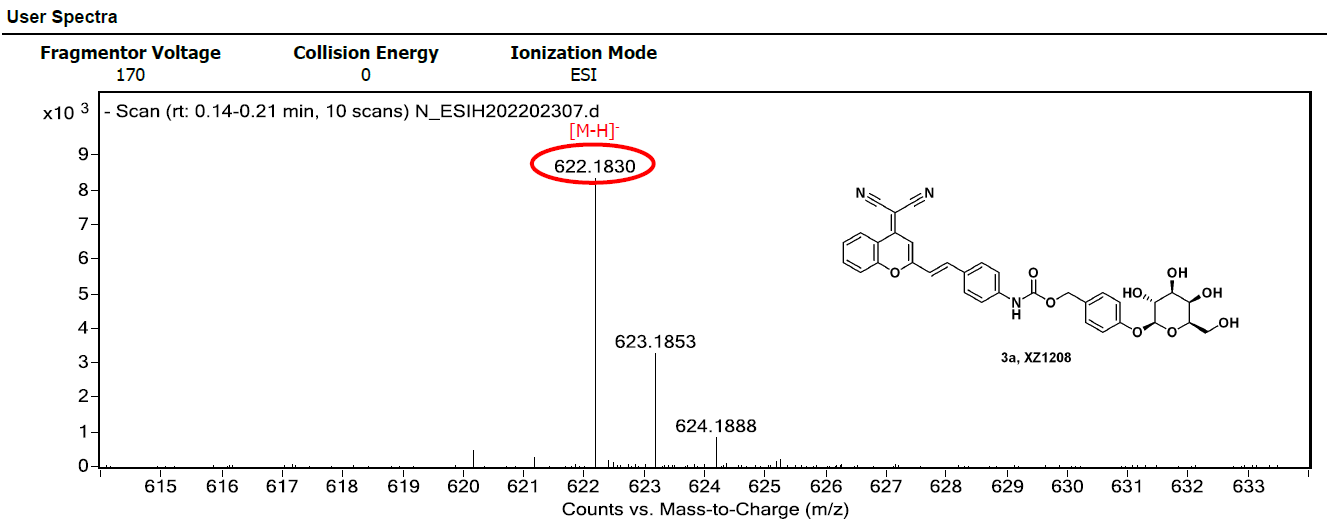


**Fig. Note 18** HRMS spectrum of **XZ1208**.


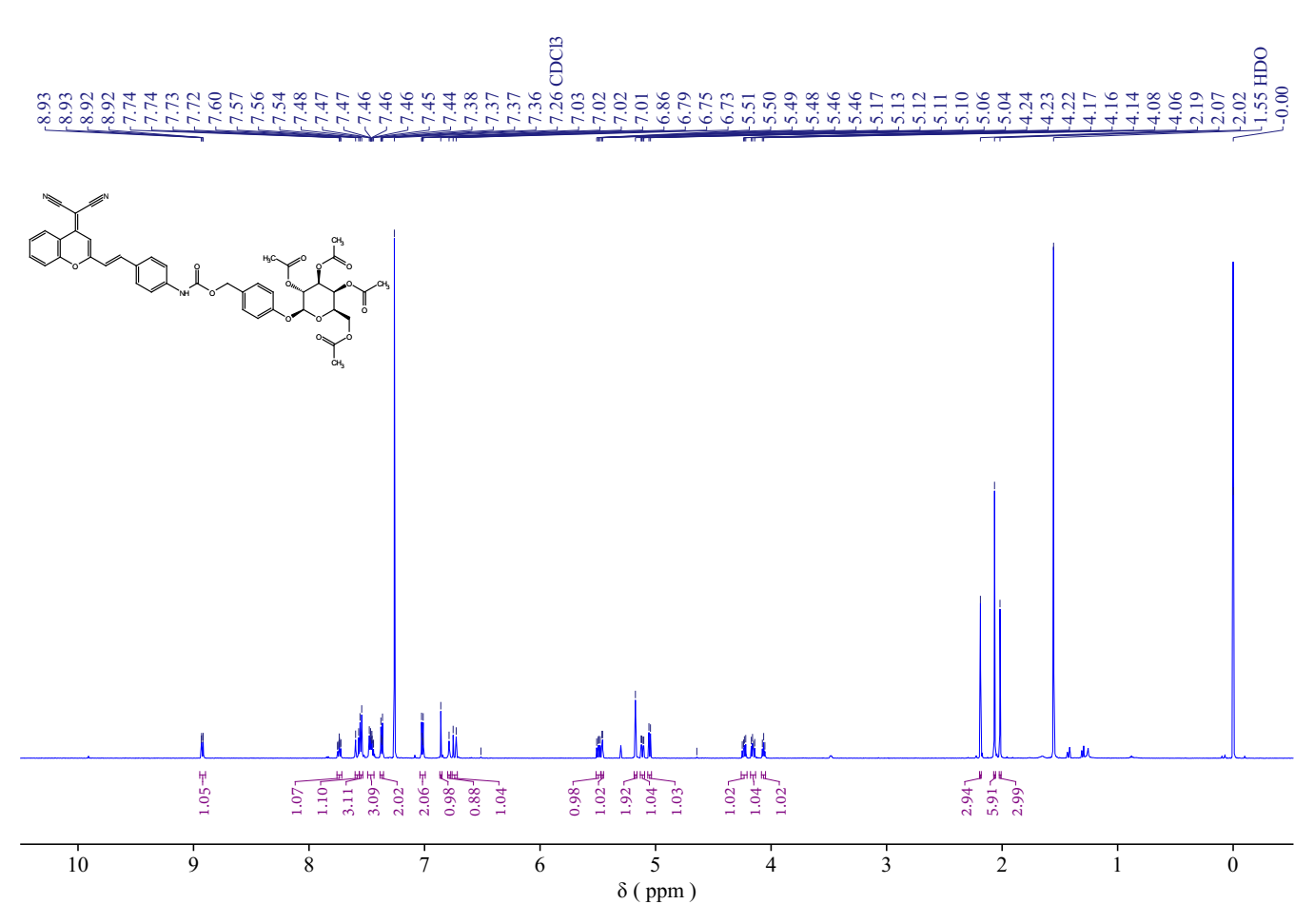


**Fig. Note 19** ^1^H-NMR spectrum of compound **3b**.


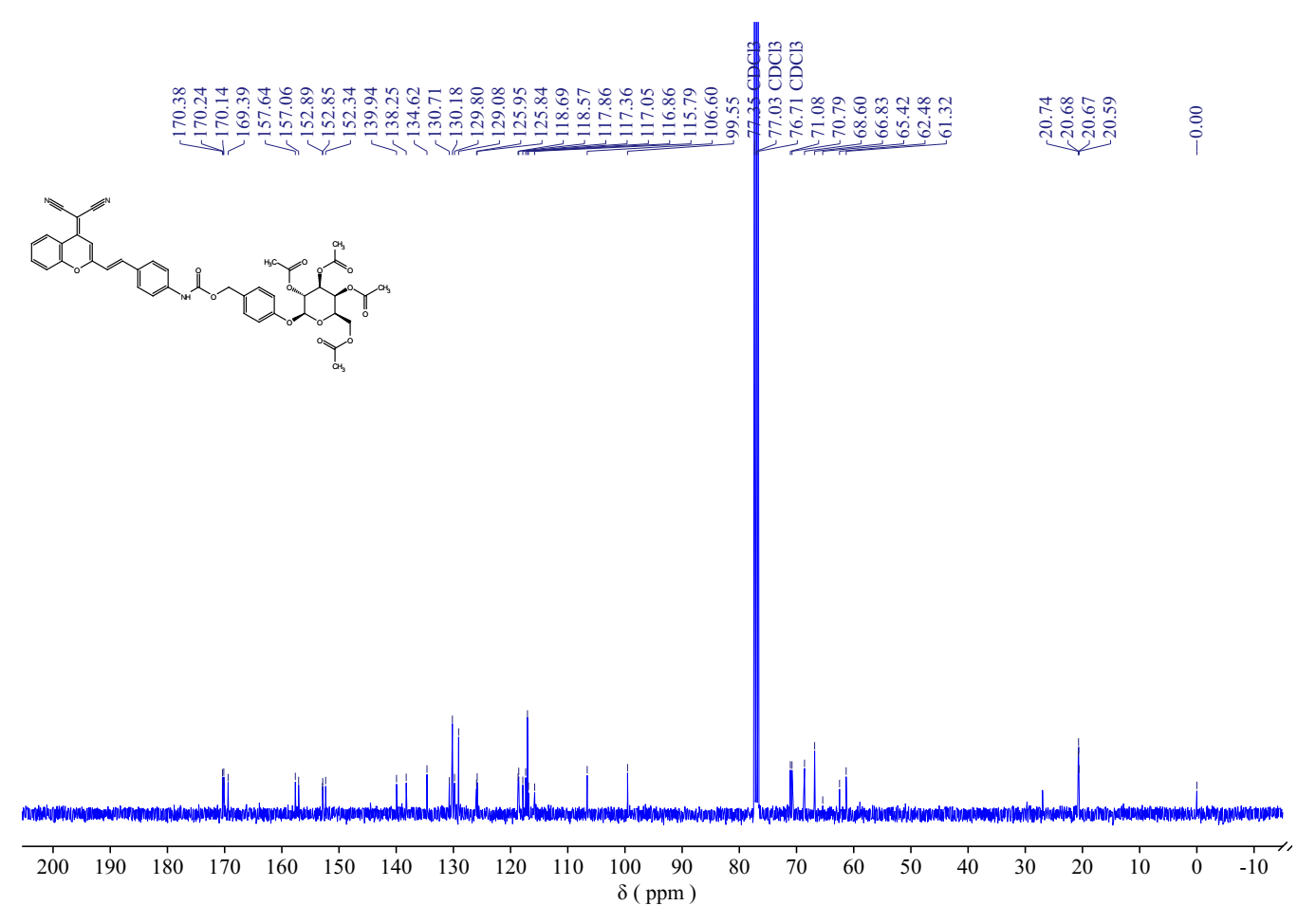


**Fig. Note 20** ^13^C-NMR spectrum of compound **3b**.


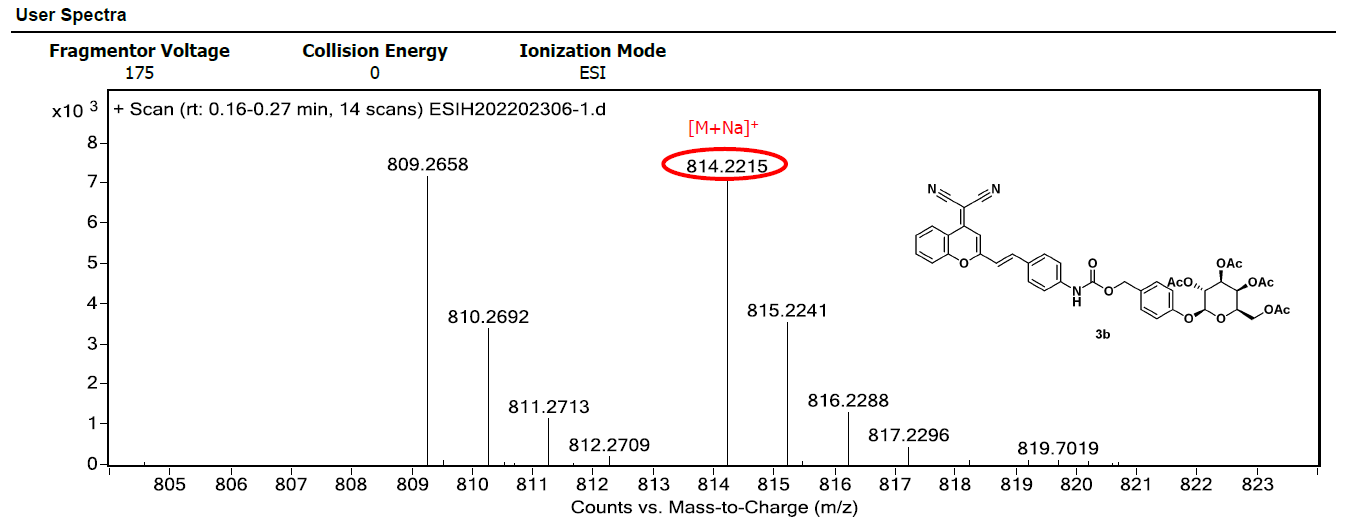


**Fig. Note 21** HRMS spectrum of compound **3b**.

**Reference**

Parker, C. A., & Rees, W. T. (1960). Correction of fluorescence spectra and measurement of fluorescence quantum efficiency. *Analyst*, *85*(1013), 587–600. https://doi.org/10.1039/AN9608500587
